# Syntheses of LSD1/HDAC Inhibitors with Demonstrated Efficacy against Colorectal Cancer: *In Vitro* and *In Vivo* Studies Including Patient-Derived Organoids

**DOI:** 10.1021/acs.jmedchem.4c01098

**Published:** 2024-09-25

**Authors:** Po-Yu Chou, Mei-Jung Lai, Kelvin K. Tsai, Li-Hsin Cheng, Yi-Wen Wu, Mei-Chuan Chen, Shiow-Lin Pan, Hsiu-O Ho, Kunal Nepali, Jing-Ping Liou

**Affiliations:** † School of Pharmacy, College of Pharmacy, Taipei Medical University, Taipei 110, Taiwan; ‡ TMU Research Center for Drug Discovery, Taipei Medical University, Taipei 110, Taiwan; § Laboratory of Advanced Molecular Therapeutics, Graduate Institute of Clinical Medicine, College of Medicine, Taipei Medical University, Taipei 110, Taiwan; ∥ Organoids Technology Core, Taipei Medical University, Taipei 110, Taiwan; ⊥ Graduate Institute of Cancer Biology and Drug Discovery, College of Medical Science and Technology, Taipei Medical University, Taipei 110, Taiwan; # TMU Research Center of Cancer Translational Medicine, Taipei 110, Taiwan; ∇ Ph.D. Program in Drug Discovery and Development Industry, College of Pharmacy, Taipei Medical University, Taipei 110031, Taiwan; ○ Clinical Drug Development of Herbal Medicine, College of Pharmacy, Taipei Medical University, Taipei 110, Taiwan; ◆ Traditional Herbal Medicine Research Center of Taipei Medical University Hospital, Taipei 110, Taiwan; ¶ Ph.D. Program for Cancer Molecular Biology and Drug Discovery, College of Medical Science and Technology, Taipei Medical University, Taipei 110, Taiwan

## Abstract

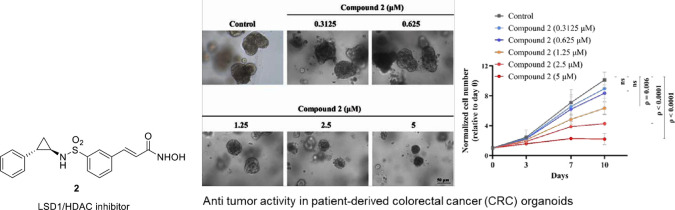

Precedential evidence ascertaining the overexpression of LSD1 and HDACs in colorectal cancer spurred us to design a series of dual LSD1-HDAC inhibitors. Capitalizing on the modular nature of the three-component HDAC inhibitory model, tranylcypromine as a surface recognition motif was appended to zinc-binding motifs via diverse linkers. A compendium of hydroxamic acids was generated and evaluated for *in vitro* cytotoxicity against HCT-116 cells (human colorectal cancer cell lines). The most potent cell growth inhibitor **2** (GI_50_ = 0.495 μMm HCT-116 cells) shows promising anticancer effects by reducing colony formation and inducing cell cycle arrest in HCT-116 cells. It exhibits preferential inhibition of HDAC6, along with potent inhibition of LSD1 compared to standard inhibitors. Moreover, Compound **2** upregulates acetyl-tubulin, acetyl-histone H3, and H3K4me2, indicative of LSD1 and HDAC inhibition. *In vivo*, it demonstrates significant antitumor activity against colorectal cancer, better than irinotecan, and effectively inhibits growth in patient-derived CRC organoids.

## Introduction

Colorectal cancer (CRC) is one of the leading causes of cancer deaths in the world.^1^ Despite a better understanding of the mechanisms contributing to the progression of CRC and significant advancement in anticancer treatment strategies to attain long-term survival, chemotherapy has not been able to yield satisfactory outcomes, and surgery remains the most effective treatment modality for CRC patients. As such, chemotherapy with 5-fluorouracil is effective in CRC; however, lack of selectivity toward the CRC cells, leading to dose-limiting toxicities, has been encountered with its use. In addition, the development of resistance of the CRC cells toward the chemotherapeutics is also a major issue, indicating the need to initiate explorations to develop targeted agents for CRC.^2^ Although earlier considered a genetic disease, recent disclosures ascertaining that the disruption of epigenetic processes leads to malignant cellular transformation have underscored the role of epigenetic aberration in CRC.^3^ The aforementioned revelation has put the components of epigenetic machinery (including DNA methylation, histone modifications, nucleosome positioning, and noncoding RNAs) in the spotlight as logical targets for anti-CRC drug discovery.^4^ Thus, inhibiting epigenetic targets is presently conceived as an effective strategy for treating CRC.

The recent progress chart of epigenetic inhibitors reveals a transposition of the medicinal chemist’s inclination from single-targeting agents to dual-targeting scaffolds. This transposed inclination of the researchers can be attributed to the mechanism of malignant diseases that suffer from complex subnetworks within the interactome involved in multiple dysregulated signaling pathways in cancer.^5^ Multitargeting agents can attenuate multiple dysregulated signaling in cancer and also outwit the limitations of conventional single-target agents, such as insufficient efficacy and development of resistance. In comparing multitargeting agents with combination therapy that works on the same principle of addressing more than one target, the former scores over the latter in terms of pharmacokinetic simplicity.^6^ Given the aforementioned, attaining an amplified therapeutic response in CRC via simultaneous or concomitant modulation of two targets is envisioned as a sagacious approach. Notably, assembling a chemical probe endowed with dual modulatory ability requires identifying the most suited combination of targets. Reliance on key revelations from the biologist ascertaining the crosstalks between the two targets or outcomes of combination therapy at the clinical level serves as the inception point of the drug discovery program centered on the design of dual inhibitory structural templates.

**Figure 1:**
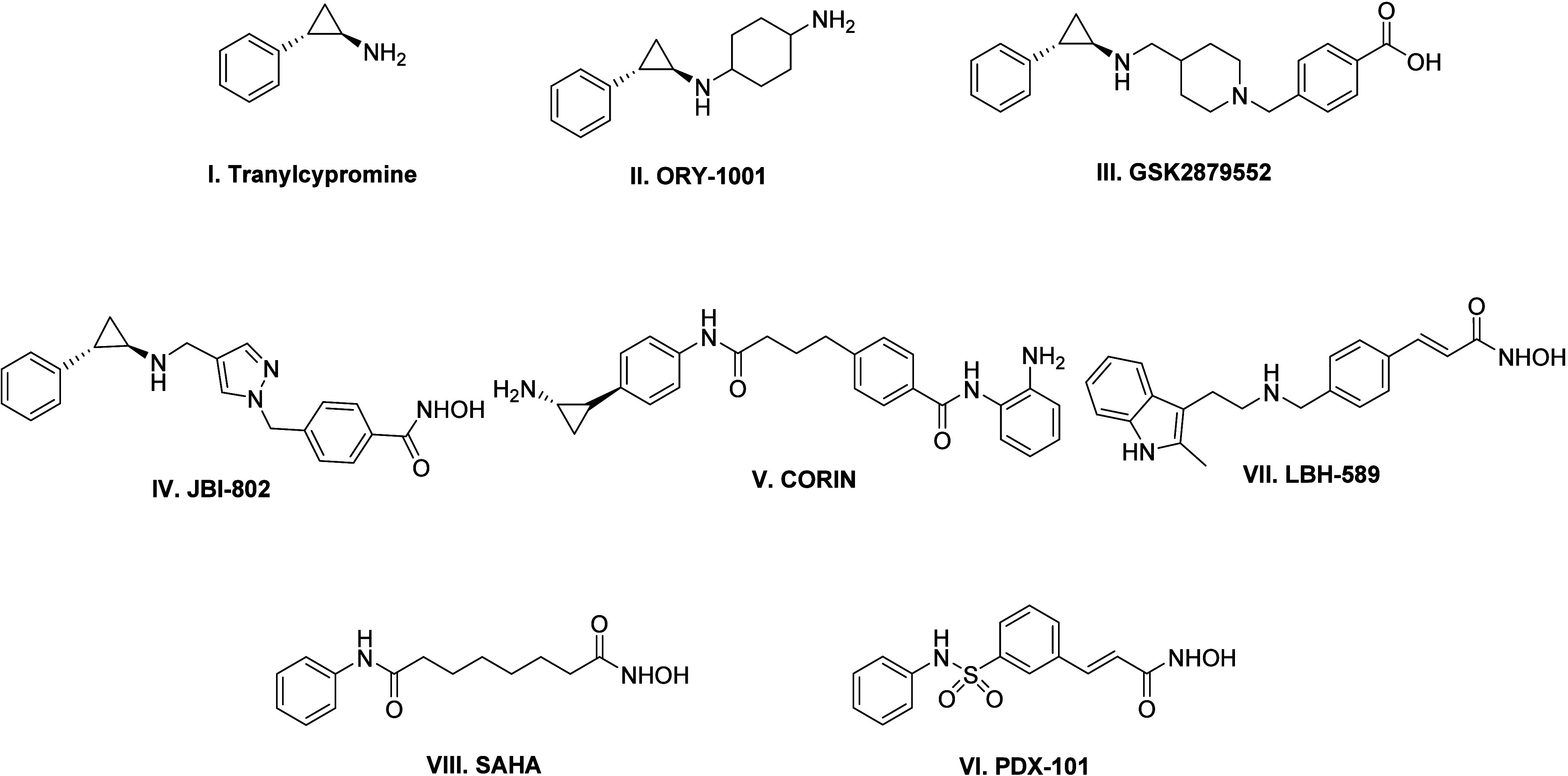
Structures of LSD1 inhibitors, dual LSD1-HDAC inhibitors, and HDAC inhibitors.

Lysine-specific demethylase 1 (LSD1/KDM1A) and histone deacetylases (HDACs) have been exhaustively explored as epigenetic targets for the design of cancer therapeutics in the past decade. As such, LSD1 behaves as either a repressor or an activator of gene expression as it removes the methyl groups from mono- and dimethyl lysine 4 or lysine 9 of histone 3 (H3K4me1/2 and H3K9me1/2).^7,8^ HDACs regulate gene expression by deacetylation of lysine residues on histone and nonhistone proteins. Deregulation of HDACs by abnormal expression or activity and the involvement of oncogenic HDAC-containing transcriptional complexes has been evidenced in diverse malignancies.^9^ Notably, both LSD1 and HDAC can be addressed simultaneously to attain synergistic efficacy owing to the functional link between the two, and this disclosure has been a catalyst to the embarkment of numerous campaigns focusing on the design of dual LSD1-HDAC modulatory structural templates.^10−12^ The magnificent antitumor efficacy of dual HDAC/LSD1 inhibitors, viz., Corin (Figure 1) targeting the corest complex (includes HDAC1 or its close paralog HDAC2, the scaffolding protein CoREST, and LSD1) to exert antitumor effects against several melanoma cell lines, 4-SC-202 manifesting antitumor activities in multiple cancer cell lines, and JBI-802 endowed with striking efficacy in AML models, highlights the success of the aforementioned campaigns.^13−15^

Literature precedents also indicate that LSD1 and HDAC are overexpressed in CRC.^16,17^ Some key revelations ascertaining the involvement of LSD1 in CRC are as follows: (i) mediation of AKT activity and promotion of epithelial-to-mesenchymal transitions by LSD1 in PIK3CA mutant CRC;^18^ (ii) the persistence of enteroendocrine progenitors mediated by LSD1, which supports *BRAF* mutant CRC;^19^ (iii) contribution of LSD1 to colon cancer metastasis via downregulation of CDH-1 expression;^20^ and (iv) promotion of secretory cell specification by LSD1 to drive BRAF mutant CRC.^21^ Also, the downregulation of specific HDACs by several types of HDAC inhibitors (pan HDAC, HDAC1,2, and selective HDAC6 inhibitors) leads to the inhibition of colon cancer cell growth.^22−26^ Thus, the findings confirming the overexpression of LSD1 and HDACs in CRC coupled with optimistic antitumor profiles of dual LSD1-HDAC inhibitors indicate that the balanced modulation of LSD1 and HDAC via dual-targeting chemical probes is a compelling tactic to attain amplified antitumor effects in CRC.

### Rational Design

The quest to develop anti-CRC interventions spurred us to embark on a drug discovery program to design structural templates that can simultaneously inhibit HDAC isoforms and LSD1. It is noteworthy that the HDAC inhibitory template, owing to its structural plasticity, has been a precursor for numerous bifunctional chemical architectures. As such, the template comprises a pragmatic appendage of three components, viz., CAP, linker, and zinc-binding motif, where the surface recognition group (CAP) interacts with the amino acid residues around the entrance of the active site, a hydrophobic linker occupies the tunnel of the active site, and a zinc-binding group chelates the catalytic zinc ion.^27^ A glance at the chemical toolbox of bifunctional inhibitors originating from the HDAC inhibitory pharmacophore highlights the remarkable accommodative ability of this three-component model to recruit diverse antitumor pharmacophores as CAP groups to direct the efficacy of the resulting scaffolds toward a specific malignancy. Thus, stitching of the LSD1 inhibitory pharmacophore as a CAP construct to the zinc-binding motif via chemically diverse linkers was envisioned as the pragmatic approach to furnish anti-CRC adducts for this study. Thus, an LSD1 inhibitory fragment was sought, and an extensive literature survey led us to conceive that tranylcypromine (an LSD1 inhibitor, Figure 1) can be easily installed within the HDAC inhibitory pharmacophore. In addition to the suitability of the chemical architecture of tranylcypromine to fit in as the surface recognition part of the HDAC inhibitor three-component model, the success of tranylcypromine-based LSD1 inhibitors (structures I–V, Figure 1) as antitumor agents at the clinical level further convinced us toward its inclusion in the assemblage of the target bifunctional adducts.^28^

**Figure 2:**
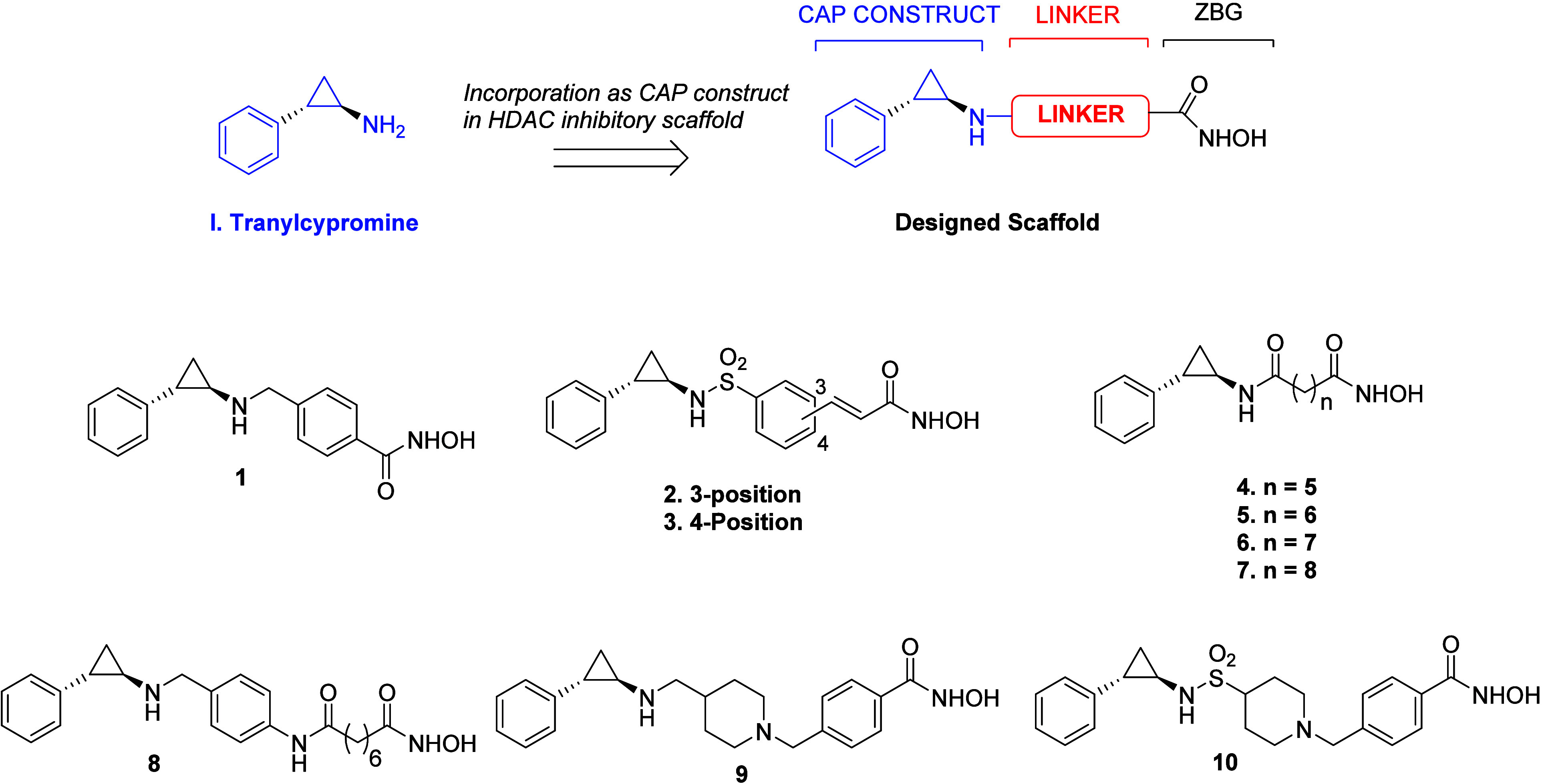
Design strategy.

With this background, tranylcypromine-appended hydroxamic acids were designed in this study (design strategy depicted in Figure 2). Notably, metal chelating hydroxamic acid functionality owing to its precedential clinical success [(SAHA, PXD101, and LBH-589) (Figure 1)] was selected for the design of target structural frameworks despite the existence of diverse types of zinc-binding motifs. Thus, tranylcypromine and hydroxamic acid functionalities were the structural attributes consistently featured in all the compounds, and the linker part was the variable component in the template of the designed compounds. Accordingly, a compendium of tranylcypromine-based HDAC inhibitors was furnished and evaluated for antiproliferative effects. As a result, a potent dual inhibitory adduct endowed with magnificent anticolorectal effects (*in vitro* and *in vivo*) was identified.

## Results and Discussion

### Chemistry

**Scheme 1:**
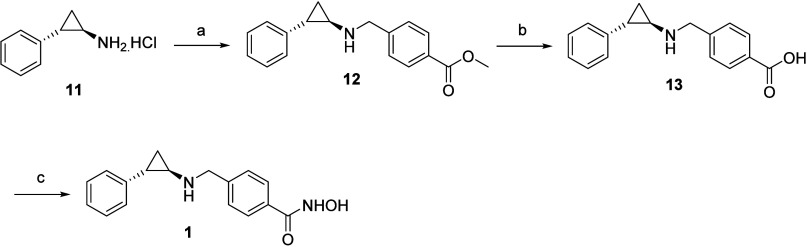
Reagents and Conditions for (a) Methyl 4-Bromobenzoate, K_2_CO_3_, DMF, RT; (b) LiOH (aq), Dioxane, RT; and (c) (i) NH_2_OTHP, EDC, HOBt, DIPEA, DMF, RT; (ii) 10% TFA (aq), MeOH, RT

**Scheme 2:**
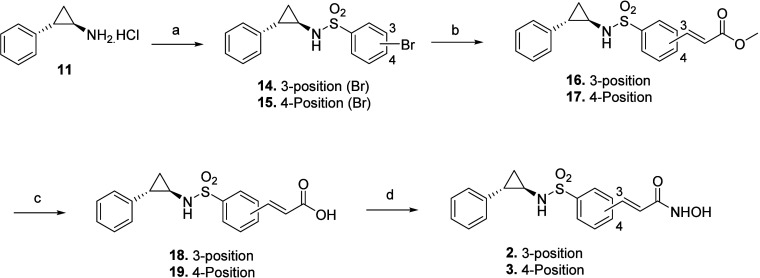
Reagents and Conditions (a) for **14**, 3-Bromobenzenesulfonyl Chloride, TEA, DCM, RT, and for **15**, 4-Bromobenzenesulfonyl Chloride, TEA, DCM, RT; (b) Pd(OAc)_2_, Triphenyl Phospine, TEA, DMF, 100° C; (c) LiOH (aq), Dioxane, RT; (d) (i) NH_2_OTHP, EDC, HOBt, DIPEA, DMF, RT; (ii) 10% TFA (aq), MeOH, RT

**Scheme 3:**
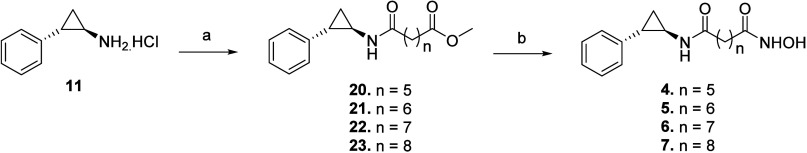
Reagents and Conditions for (a) Alkoxy Alkanoic Acids, EDC, HOBt, DIPEA, DMF, RT; (b) (i) NH_2_OTHP, EDC, HOBt, DIPEA, DMF, RT; (ii) 10% TFA (aq), MeOH, RT

**Scheme 4:**
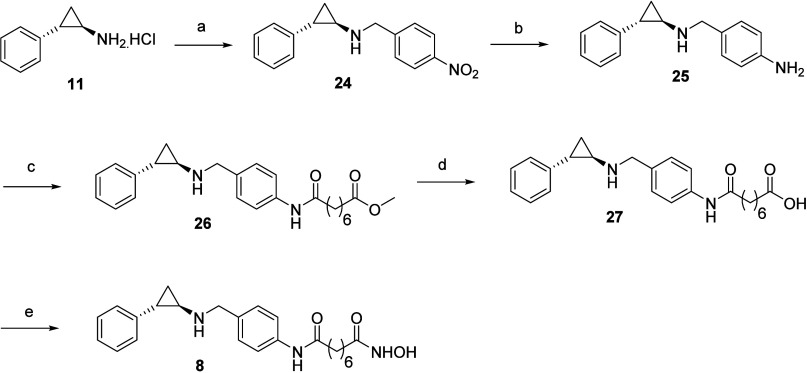
Reagents and Conditions for (a) 1-(Bromomethyl)-4-nitrobenzene, K_2_CO_3_, DMF, RT; (b) Fe, NH_4_Cl, C_2_H_5_OH: H_2_O:: 9:1, reflux; (c) Monomethyl Suberate, EDC, HOBt, DIPEA, DMF, RT; (d) LiOH (aq), Dioxane, RT; (e) (i) NH_2_OTHP, EDC, HOBt, DIPEA, DMF, RT; (ii) 10% TFA (aq), MeOH, RT

**Scheme 5:**
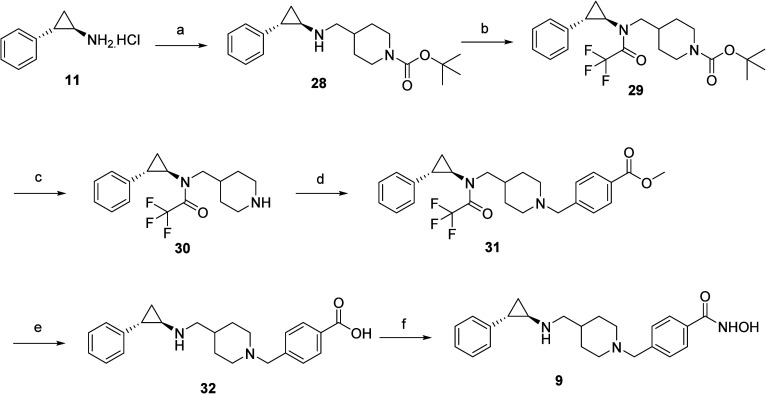
Reagents and Conditions for (a) (i) NH_4_OH, H_2_O, RT; (ii) *tert*-Butyl 4-Formylpiperidine-1-carboxylate, NaBH(OAC)_3_, CH_3_COOH, DCE, RT; (b) Trifluoroacetic Anhydride, DCM, RT; (c) Trifloroacetic Acid, DCM, RT; (d) Methyl 4-(Bromomethyl)benzoate, K_2_CO_3_, DMF, RT; (e) NaOH (50% aq), Methanol, RT; (f) (i) NH_2_OTHP, EDC, HOBt, DIPEA, DMF, RT; (ii) 10% TFA (aq), MeOH, RT

**Scheme 6:**
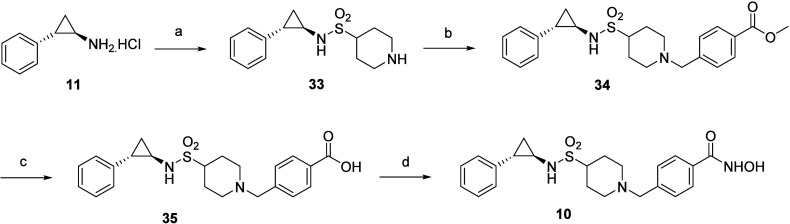
Reagents and Conditions for (a) (i) *tert*-Butyl 4-(Chlorosulfonyl)piperidine-1-carboxylate, TEA, DCM, RT; (ii) TFA (neat), DCM, RT; (b) Methyl 4-(Bromomethyl)benzoate, K_2_CO_3_, DMF, RT; (c) LiOH (aq), Dioxane, RT; (d) (i) NH_2_OTHP, EDC, HOBt, DIPEA, DMF, RT; (ii) 10% TFA (aq), MeOH, RT

The multistep synthetic routes optimized for synthesizing target compounds **1**–**10** are depicted in Schemes 1, 2, 3, 4, 5, and 6. Target adduct **1** was furnished via a sequence of reactions shown in Scheme 1, which commenced with the appendage of the starting material **11** with methyl 4-(bromomethyl)benzoate using potassium carbonate as the base. Notably, despite the presence of primary amine functionality in **11**, selective formation of the monobenzylated product was observed at room temperature. Subjection of intermediate **12** to lithium hydroxide-mediated ester hydrolysis yielded carboxylic acid **13,** which on EDC/HOBT assisted amidation with NH_2_OTHP followed by subsequent cleavage of the tetrahydropyranyl moiety via protic acid culminated in the identification of hydroxamic acid **1**.

The benzenesulfonyl acrylamide bearing adducts, **2** and **3**, were generated via a chemical route illustrated in Scheme 2. The route began with the sulfonylation of **11** with 3-bromobenzenesulfonyl chloride and 4-bromobenzenesulfonyl chloride to obtain sulfonamides **14** and **15.** Heck olefination was carried out to accomplish C–C bond coupling to produce the α,β-unsaturated esters **16** and **17.** Ester hydrolysis followed by amidation with NH_2_OTHP and trifluoroacetic acid-mediated cleavage of tetrahydropyranyl functionality led to the synthesis of the designed compounds **2** and **3**.

The reaction sequence to furnish the desired methylene chain bearing hydroxamic acids **4**–**7** is outlined in Scheme 3. Alkoxy alkanoic acids of varied lengths were used for the amidation of **11**, employing the carbodiimide-based methodology. The resulting intermediates were converted to hydroxamic acids using the same method described in Schemes 1 and 2.

Scheme 4 presents the synthetic pathway to a lengthy linker-bearing hydroxamic acid **8**. Benzylation of **11** with 1-(bromomethyl)-4-nitrobenzene using potassium carbonate as the base yielded intermediate **24**, which on reduction with Fe/NH_4_Cl generated the primary amine **25**. The primary amine was treated with monomethyl suberate using the EDC/HOBt-mediated amidation protocol to obtain adduct **26**. Adduct **26** was subjected to the reaction sequence, viz., ester hydrolysis, amidation with NH_2_OTHP, and protic acid-mediated cleavage of the tetrahydropyranyl moiety to furnish compound **8**.

The multistep synthetic route employed for synthesizing target compound **9** is shown in Scheme 5. Starting material **11** was converted to a nonionic form and then subjected to reductive amination with *tert*-butyl 4-formylpiperidine-1-carboxylate using sodium triacetoxyborohydride as the reducing agent to furnish intermediate **28**. Protection of secondary amine functionality was carried out with trifloroacetic anhydride, and the resulting intermediate **29** was then treated with trifloroacetic acid to afford the Boc-deprotected intermediate **30**. Benzylation of intermediate **30** with methyl 4-(bromomethyl)benzoate yielded **31**, which on hydrolysis led to the furnishment of carboxylic acid **32**. The carboxylic acid **32** was converted to the hydroxamic acid **9** using the same reagents and the reaction conditions mentioned in the previous schemes.

The reaction sequence depicted in Scheme 6 was employed to replace the **″–CH_2_–″** unit of adduct **9** with an “**–SO_2_–**″ unit via synthesis of target compound **10**. The pathway commenced with the sulfonylation of **11** with *tert*-butyl 4-(chlorosulfonyl)piperidine-1-carboxylate to afford an intermediate, which was subsequently treated with TFA (neat condition) to yield the Boc-deprotected adduct **33**. Adduct **33** was exposed to the same reaction sequence employed in Scheme 6 to obtain **10.**

### Biological Evaluation

#### *In Vitro* Cytotoxicity

**Figure 3:**
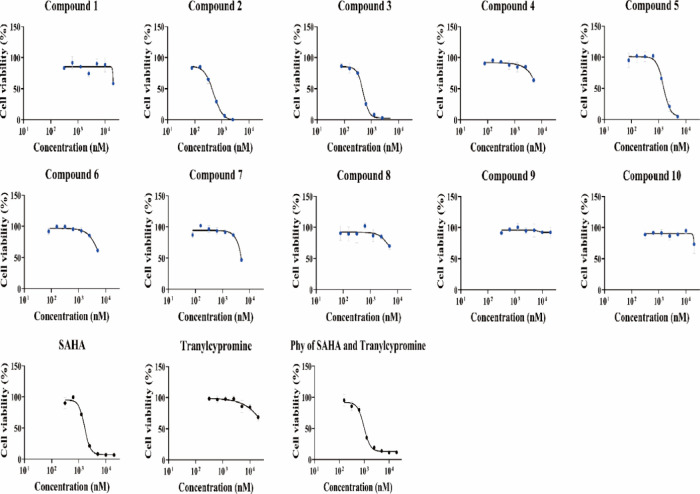
Cell growth inhibition activity of SAHA, tranylcypromine, physical mixture (phy) of SAHA and tranylcypromine, and synthetic compounds (**1**–**10**) in the colorectal HCT-116 cancer cell line. Cell viability was measured using the Cell Counting Kit-8 assay after treatment. Data are expressed as means ± SD of at least three independent experiments. Phy of SAHA and tranylcypromine, physical mixture of SAHA and tranylcypromine; SAHA, suberoylanilide hydroxamic acid (vorinostat).

An *in vitro* cytotoxicity study of the synthesized compounds against human colorectal HCT-116 cells was conducted to interrogate the impact of chemically diverse linkers on cellular activity. As shown in Figure 3 and Table 1, a comparison of the antiproliferative effects of the compounds was done with SAHA (FDA-approved HDAC inhibitor) and tranylcypromine (LSD1 inhibitor). Although SAHA elicited substantial activity against HCT-116 cell lines (GI_50_ = 1.64 μM), tranylcypromine was observed to be devoid of anti-CRC effects. Moreover, the HDAC inhibitor (SAHA) and LSD1 inhibitor (tranylcypromine) synergistically decreased cell viability and inhibited cell growth in human colorectal HCT-116 cells (GI_50_ = 0.978 μM). The results show that combined inhibition of LSD1 and HDACs is more effective than the inhibition of a single enzyme in human colorectal HCT-116 cells. The results presented in Table 1 depict that HCT-116 exhibited sensitivity to the exposure of selective hydroxamic acids. Notably, only compounds **2**, **3**, and **5** manifested significant cell growth inhibitory effects against HCT-116 cells with GI_50_ values of 0.495, 0.496, and 1.51 μM, respectively, while other compounds were devoid of antitumor effects (GI_50_ values >10 μM). These findings perspicuously depict that the linker recruitment was highly crucial for the anti-CRC effects as the chemical architecture of the designed compounds differs from each other only in terms of linkers with tranylcypromine (surface recognition part) and hydroxamic acid (zinc-binding motif) being the overlapping structural attributes in all the compounds. Encouragingly, hydroxamic acids **2**, **3**, and **5** demonstrated more pronounced cell growth inhibitory effects against CRC cell lines than the FDA-approved HDAC inhibitor (SAHA) employed as a standard in this study. Of the three compounds mentioned above, adducts 2 and 3 were strikingly cytotoxic toward HCT-116 cell lines and considered worthy enough for exhaustive evaluations. Although compounds **2** and **3** displayed equipotent antiproliferative effects against HCT-116 cell lines, compound **3** was down-prioritized for further investigations owing to its relatively low yields (Scheme 2, 41% yield) compared to compound **2** (66% yield). Another reason for prioritizing compound **2** over **3** for detailed profiling was its structural overlap with FDA-approved HDAC inhibitor LBH-589 in the context of the site of stitching of the acrylamide unit.

**Table 1:** Antiproliferative Activity of Compounds **1**–**10** against Human Colorectal Cell Lines

	GI_50_ (μM ± SD^a^)
compounds	HCT-116 cell line
**1**	>10
**2**	0.495 ± 1.04
**3**	0.496 ± 1.06
**4**	>10
**5**	1.51 ± 1.08
**6**	>10
**7**	>10
**8**	>10
**9**	>10
**10**	>10
**SAHA**	1.64 ± 1.04
**tranylcypromine**	>10
**Phy of SAHA and tranylcypromine**	0.978 ± 1.05

a SD: standard deviation. All experiments were independently performed at least three times (*n* = 3) to determine the mean and SD.

### Colony Formation in Evaluation of Antitumor Activity in Human Colorectal Cancer Cells

**Figure 4:**
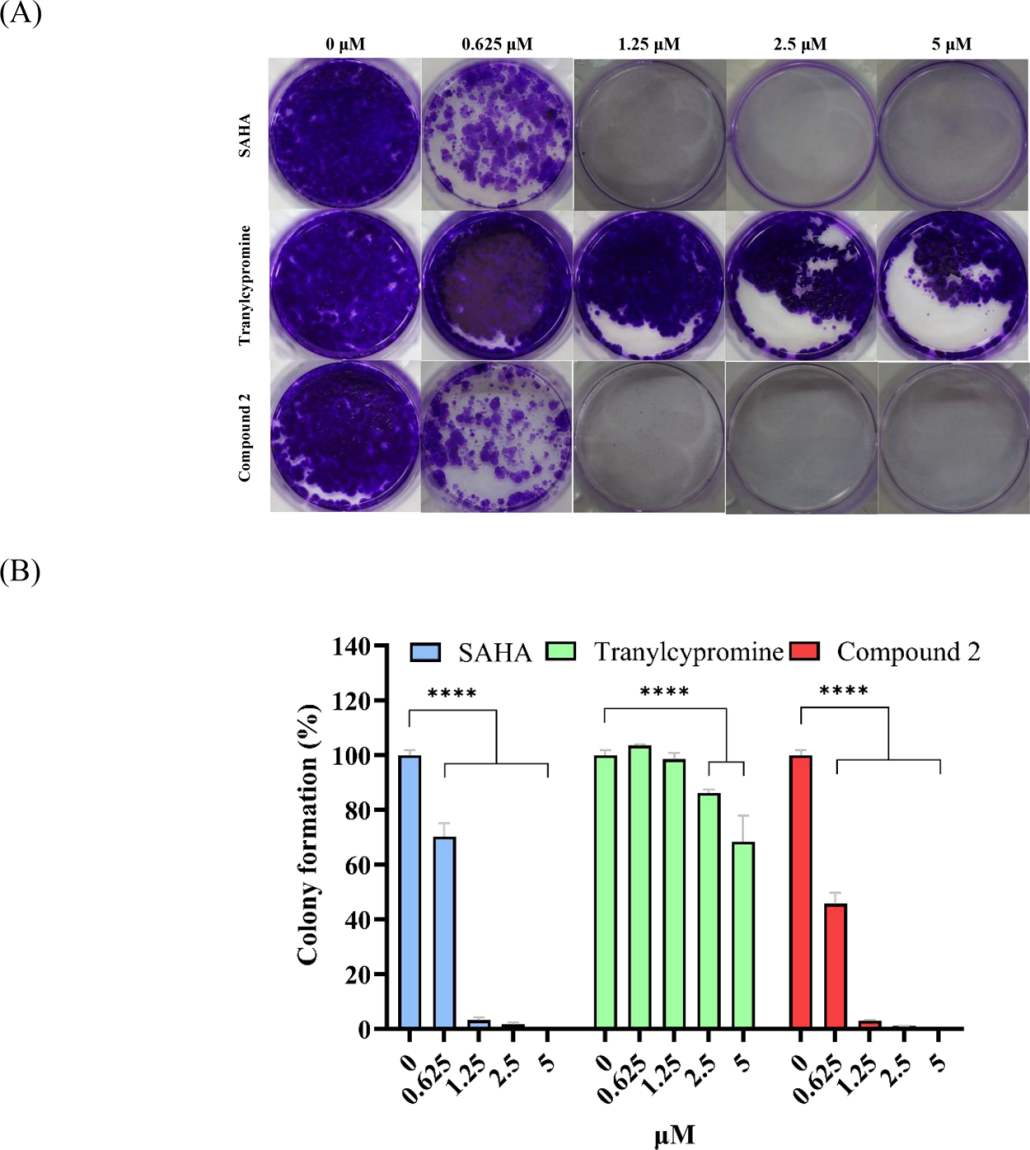
Compound **2** suppresses colony formation in colorectal HCT-116 cancer cells. Compound **2**-mediated concentration-dependent inhibition of colony formation in HCT-116 cancer cell lines (A) and quantification of colony numbers, expressed as a percentage of controls (B). Data are expressed as means ± SD of at least three independent experiments. Statistical significance in (B) is indicated by ****p < 0.0001 compared with the control group, as determined using one-way ANOVA.

The tumoricidal effects of compound **2** were further evaluated by observing its impact on the ability of human colorectal HCT-116 cells to form colonies. Encouragingly, it was found that compound **2** significantly reduced colony formation at a concentration of 0.625 μM after 3 days of treatment with HCT-116 cells (Figure 4A). Quantitative results shown in Figure 4B revealed that **2** exerted a more pronounced reduction of colony formation than SAHA at the concentration of 0.625 μM (45.71 and 70.13%, respectively). Notably, tranylcypromine treatment at the same or higher concentration exhibited no difference in the number of tumor colonies.

### Evaluation of Cell Cycle Progression and Cell Death Response

**Figure 5:**
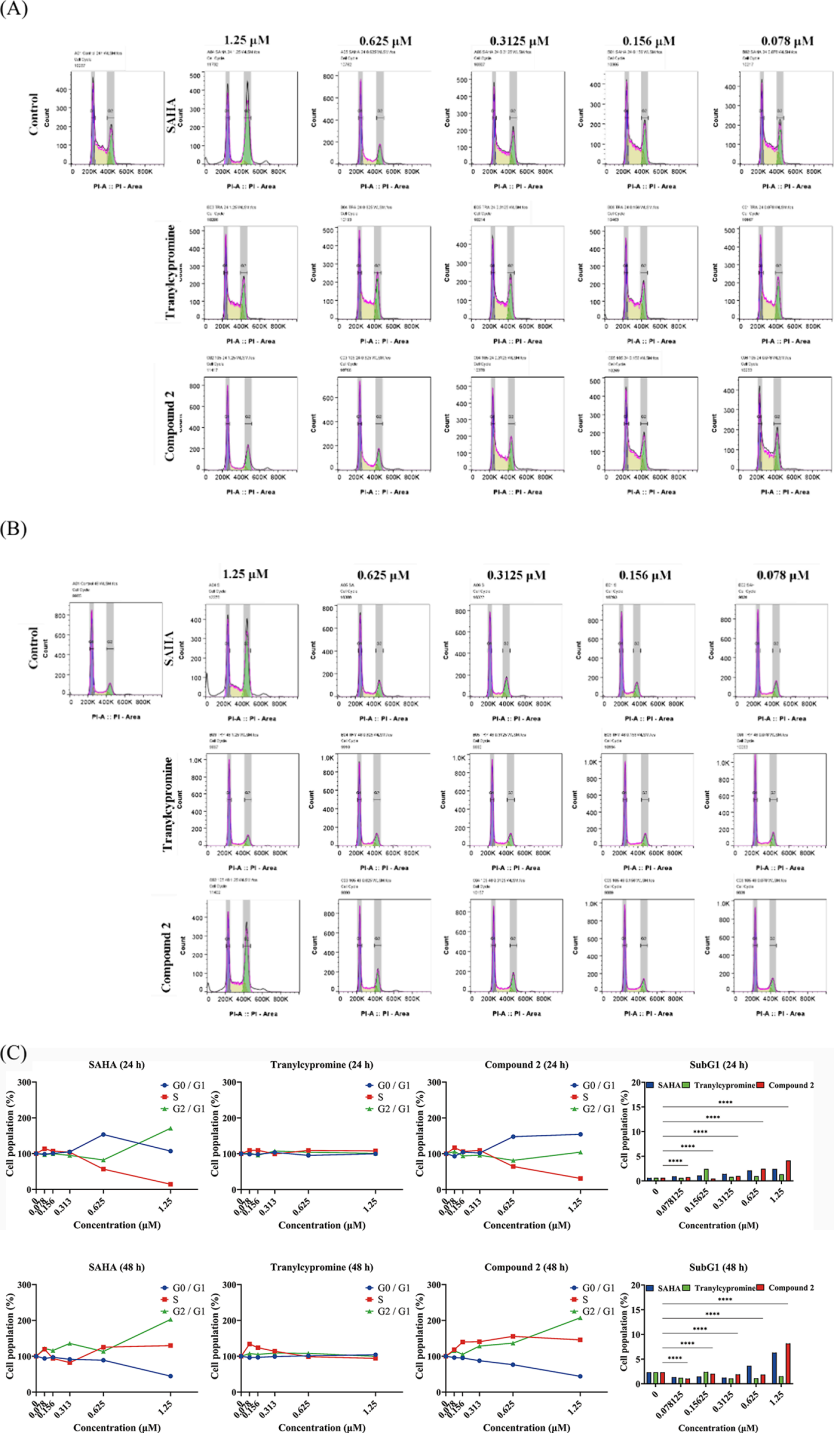
Compound **2** induces sub-G1 phase accumulation in HCT-116 cells. Concentration-dependent effects of **2** on sub-G1 phase accumulation in HCT-116 cells. Cells were treated with DMSO, SAHA, tranylcypromine, or **2** with indicated concentrations for (A) 24 h and (B) 48 h, and the cell cycle distribution was analyzed by flow cytometry. (C) The percentages of the G0/G1, S, G2/M, and sub-G1 phases were quantified. Data are expressed as means ± SD of at least three independent experiments. Statistical significance in (B) is indicated by ****p < 0.0001 compared with the control group, as determined using one-way ANOVA.

The correlation of cell proliferation with cell cycle control and progression is well established.^29^ Flow cytometric analysis was performed to assess the cell cycle distribution in HCT-116 cells after treatment with the standards (HDAC and LSD1 inhibitor) and compound **2** at different concentrations for 24–48 h (Figure 5A,B). The percentages of G0/G1, S, and G2/M phases and sub-G1 phases (Figure 5C) were quantified. No substantial difference in the percentages of cell cycle phases following different concentrations of tranylcypromine treatment for 24–48 h compared with the DMSO control (Figure 5A–C was observed. However, treatment with higher concentrations of SAHA and compound **2** led to a significantly decreased cell number in the S phases at 24 h and the G0/G1 phase at 48 h. Primarily, treatment with **2** induced the sub-G1 population in a time-dependent manner at 48 h, while SAHA demonstrated weaker effects and delayed cytotoxicity. These results suggest that compound **2** exerted cell cycle arrest at the G0/G1 phase, eventually leading to cell death.

### Evaluation of HDAC Isoform Inhibition

All the compounds were assessed for their ability to inhibit HDAC isoforms (HDAC1, 2, 6, and 8). The Reaction Biology Corporation, USA, performed the HDAC inhibition assay. The HDACs 1, 2, and 6 substrate is a fluorogenic peptide from p53 residues 379–382 (RHKK(Ac)AMC). The substrate for HDAC8 is a fluorogenic peptide from p53 residues 379–382 (RHK(Ac)K(Ac)AMC). The assay results (Table 2) revealed that the compounds manifested a varied inhibitory profile against the HDAC isoforms. Notably, the installation of diverse linkers in the structural template of the designed compounds influenced the HDAC inhibitory effects of the compounds. In general, the HDAC6 isoform was the most sensitive to the exposure of the synthesized compounds **1**–**10**. Thus, few notions regarding the linker-HDAC6 inhibitory activity were deduced. It was observed that the placement of benzenesulfonyl acrylamide and methylene chain-type linkers was beneficial for HDAC inhibitory activity of the compounds. Benzenesulfonyl acrylamide linker-bearing compounds **2** and **3** elicited striking HDAC6 inhibitory effects with IC_50_ values of 11 and 9 nM, respectively. Methylene chain linker-containing compounds (**4**–**7**) also exerted significant HDAC6 inhibition with IC_50_ values of **27**, **9**, **17**, and **18** nM, respectively. It is noteworthy to mention that among the methylene-type linker-bearing compounds, compound **5**, which is composed of the same number of methylene units as present in SAHA, was the most potent against HDAC6. The tactic of increasing the length of the linker exhibited a variable impact on the HDAC6 inhibitory effects, with only compound **10** bearing a sulfonyl piperidine linker demonstrating an impressive HDAC6 inhibitory activity at a single-digit nanomolar concentration (IC_50_ value = 9 nM). The other two compounds with lengthy linkers (**8** and **9**) were significantly inferior to compounds **2**–**7** as HDAC6 inhibitors. All the potent HDAC6 inhibitors **2**, **3**, **4**, **5**, **6**, **7**, and **10** also exerted inhibitory activity against HDAC1, 2, and HDAC8 isoforms; however, the compounds were many folds more preferential toward the HDAC6 isoform.

**Table 2:** HDAC Inhibition Activity of Compounds^a^

	**IC**_**50**_**(nM)**^b^			
**compounds**	**HDAC1**	**HDAC2**	**HDAC6**	**HDAC8**
**1**	8.72		0.821	
**2**	0.125	0.373	0.0118	0.103
**3**	0.116	0.343	0.00966	0.753
**4**	0.795	2.27	0.0277	1.29
**5**	0.105	0.239	0.00966	0.511
**6**	0.0817	0.372	0.0171	0.575
**7**	0.236	0.766	0.0189	0.473
**8**			3.73	
**9**	12.9		0.100	1.26
**10**	4.12	8.30	0.00932	0.407
**SAHA**	0.0424	0.179	0.00214	0.791
**Tranylcypromine**				
Trichostatin A	0.0014	0.00456	0.000712	0.253

a Empty cells indicate no inhibition observed or compound activity that could not be fit to an IC_50_ curve.

b This assay was conducted by the Reaction Biology Corporation, Malvern, Pennsylvania. Compounds were dissolved in DMSO and tested in 10-dose IC_50_ mode with threefold serial dilution starting at 10 μM.

### Evaluation of LSD1 Inhibition

The synthesized compounds were evaluated for their ability to inhibit LSD1 by the fluorescence coupling enzyme assay. The compounds were submitted to Reaction Biology Corporation (Malvern, Pennsylvania). For the fluorescence coupling enzyme assay, the production of FAD-dependent H_2_O_2_ as a result of demethylase activity of LSD1 was measured. Tranylcypromine and SAHA were employed as standards for the comparative analysis. The results, as depicted in Table 3, indicate that the linker used for the appendage of tranylcypromine with the hydroxamic acid functionality had a significant impact on the LSD1 inhibitory activity. Compound **1** featuring a benzyl linker elicited poor LSD1 inhibitory effects (IC_50_ = 28 μM) and was around twofold less potent in inhibiting the enzyme than tranylcypromine. The benzene sulfonyl acrylamide (linker) installation in the chemical architecture of the designed compounds led to favorable LSD1 inhibitory activity as compounds **2** and **3** exerted LSD1 inhibition with IC_50_ values of 0.571 and 0.899 μM, respectively. Both the compounds displayed significantly more pronounced LSD1 inhibitory effects (>15 folds) than tranylcypromine. Notably, the site of stitching the acrylamide functionality on the benzene sulfonyl part (compounds **2** and **3**) also led to differential LSD1 inhibitory activity. Compound **2**, with acrylamide functionality tethered at the meta position, was around 1.5 folds more potent than compound **3** (acrylamide placed at the para position). Unlike the HDAC isoform inhibitory assay (Table 2), where methylene unit-based linker-bearing compounds exerted substantial HDAC6 inhibition, placement of long methylene chain linkers did not yield favorable trends in LSD1 inhibitory assay, and compounds **4**–**7** demonstrated poor to moderate LSD1 inhibitory potential. The linker lengthening approach proved beneficial as compounds **8**, **9**, and **10** were endowed with significant LSD1 inhibitory effects with IC_50_ values in the low μM concentration range; however, none of these compounds were superior to their counterparts **2** and **3** as LSD1 inhibitors. Overall, compound **2** was found to be the most potent LSD1 inhibitor.

**Table 3:** LSD1 Inhibition Activity of Compounds

**compounds**	**IC**_**50**_**(μM, LSD1 inhibition)**^a^
**1**	28
**2**	0.571
**3**	0.899
**4**	38.3
**5**	14.6
**6**	17.10
**7**	7.68
**8**	1.61
**9**	0.958
**10**	2.40
**SAHA**	106
**Tranylcypromine**	14.8

a This assay was conducted by the Reaction Biology Corporation, Malvern, Pennsylvania. Compounds were tested in 10-dose IC_50_ mode, in a singlet, with threefold serial dilution starting at 100 μM.

**Figure 6:**
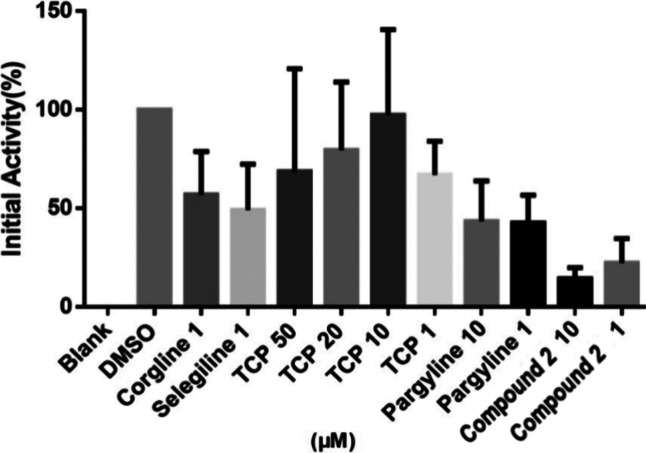
LSD1 enzyme inhibitory activity of compound **2**.

Compound **2**, being the most potent LSD1 inhibitor, was further assessed to inhibit the activity of LSD1 at 1 and 10 μM via the KDM1/LSD1 activity quantification/colorimetric assay. A comparison of the LSD1 inhibitory activity of **2** was done with Corgline, selegiline, tranylcypromine, and pargyline (standard LSD1 inhibitor). The outcome of this study led to optimistic revelations as the hydroxamic acid **2**, even at 1 μM, was found to be more effective in inhibiting the activity of LSD1 than the standard LSD1 inhibitor at all the concentrations tested. The correlation of results depicted in Tables 1–3 and Figure 6 advocates for dual LSD1/HDAC6 modulation as the underlying mechanism for the anti-CRC effects of compound **2**.

### Western Blot Analysis for HDAC Inhibition, LSD1 Inhibition, and Cell Death Response

**Figure 7:**
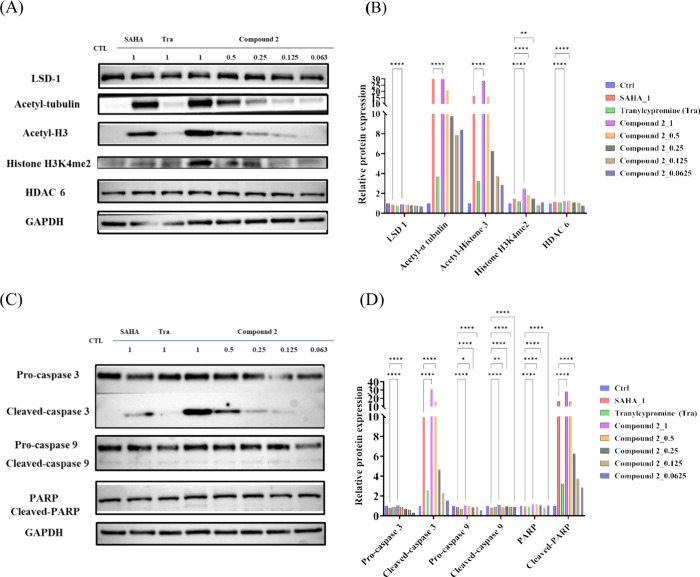
Compound **2** leads to the upregulation of acetyl-tubulin as well as acetyl-histone H3 and triggers H3K4me2 accumulation in HCT-116 cells in a concentration-dependent manner. (A) Western blot analysis and (B) semiquantification of the HDAC inhibition and LSD1 relative biomarker proteins in HCT-116 cells after treatment with compound **2**. Compound **2** induces significant apoptosis with indicated concentrations for 24 h in HCT-116 cells, and cell lysates were immunoblotted using the indicated antibodies. (C) Western blot analysis and (D) semiquantification of the apoptosis-related proteins in HCT-116 cells after treatment with compound **2**. Statistical significance in (B) and (D) is indicated by **p < 0.01 and ****p < 0.0001 compared with the SAHA group, as determined using one-way ANOVA. SAHA, suberoylanilide hydroxamic acid (vorinostat); Tra, tranylcypromine.

The impact of compound **2** treatment on the expression levels of acetyl-histone H3 and acetyl-tubulin in human CRC cells (HCT-116) [HDAC inhibition biomarkers] was assessed by Western blot analysis. As shown in Figure 7A, treatment with compound **2** resulted in a dose-dependent upregulation of both acetyl-tubulin and acetyl-histone H3. These results are in resonance with the previous studies confirming histones as widely used substrates to determine the activity of most HDACs *in vitro*.^30,31^ Also, the outcome of Western blotting analysis indicated the ability of compound **2** to induce H3K4me2 accumulation and this observation corroborates the results of previous investigations on LSD1 inhibitors in the context of increased expression level of histone H3K4 dimethylation (H3K4me2) on treatment with the LSD1 inhibitor.^32^ The highest expressions of HDAC inhibition and LSD1 relative biomarker proteins were achieved with the treatment of compound **2** at 1 μM (*p* < 0.0001) (Figure 7B). It has been reported that the regulation of SAHA and LSD1 can induce apoptosis in cancer cells. The molecular mechanism of apoptosis involves numerous proteins, with caspases playing a central role. In normal cells, caspases exist in their inactive pro-form. Upon activation, they cleave specific substrates, triggering a proteolytic cascade that leads to apoptosis. As illustrated in Figure 7C, compound **2** induced histone H3K4me2 accumulation and apoptosis in HCT-116 cells through the activation of caspase family members (caspases 3 and 9) and causing PARP cleavage. The highest expression of cleaved-caspase 3, cleaved-caspase 9, and cleaved-PARP were achieved with the treatment of compound **2** at 1 μM (*p* < 0.0001) (Figure 7D). Although the Western blot results in Figure 7A show that SAHA robustly increases the levels of histone H3K4me2, suggesting it might have an indirect LSD1 inhibitory effect. The increase in H3K4me2 levels may be a secondary effect of HDAC inhibition leading to changes in the chromatin structure and gene expression, rather than a direct inhibition of LSD1. However, SAHA is not considered a potent direct LSD1 inhibitor. The design of dual LSD1/HDAC inhibitors, compound **2**, is essential because they provide more comprehensive inhibition by directly targeting both HDAC and LSD1, as evidenced by the higher expression of apoptosis markers (H3K4me2, cleaved-caspase 3, and cleaved-PARP) and the greater inhibition of cell growth. Collectively, these findings are aligned with signatory features of *in vitro* LSD1 and HDAC inhibition (Tables 2 and Table 3 and Figure 6) and strengthens the evidence indicating the mediation of anti-CRC effects of compound **2** through dual LSD1/HDAC inhibition.

### *In Vivo* Pharmacodynamics Study of Compound **2**

**Figure 8:**
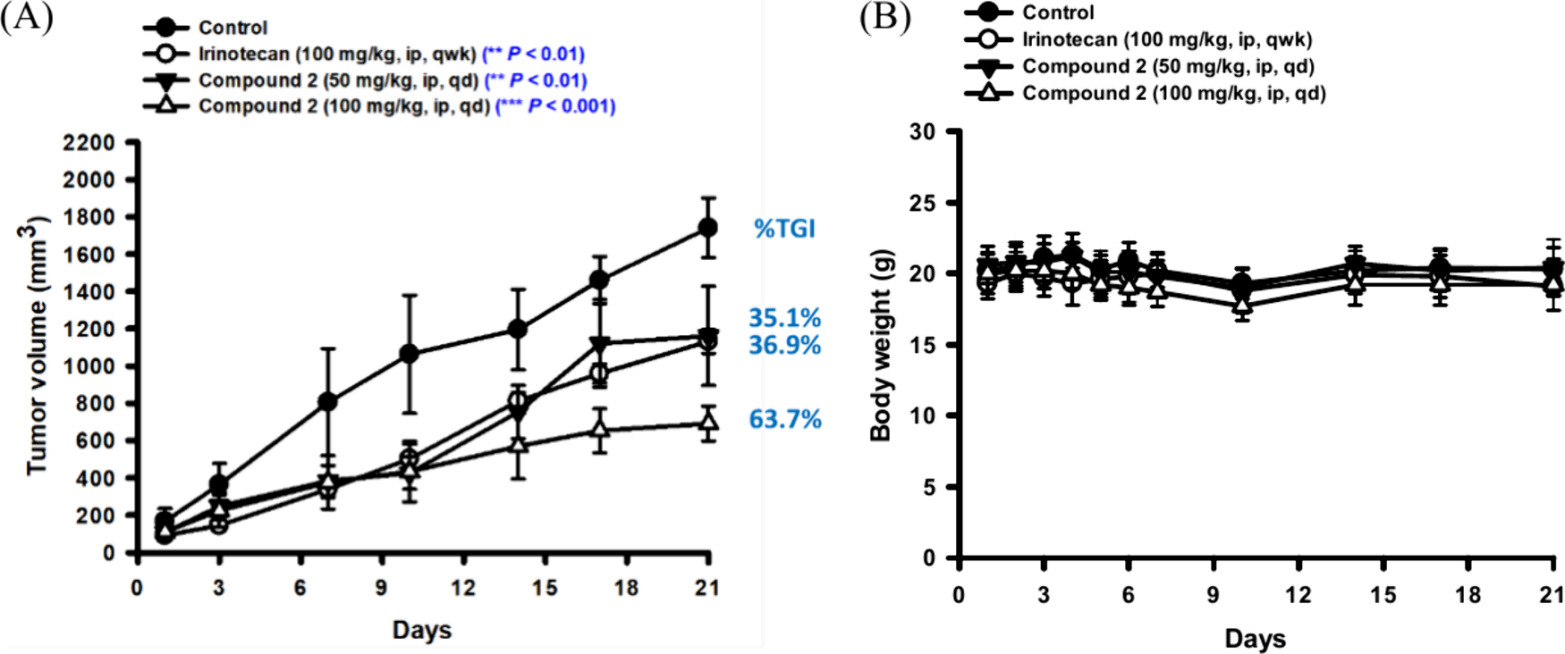
Inhibition of tumor growth by **2** treatments. The colorectal cancer cell line, HCT-116, was subcutaneously implanted in nude mice. The mice were divided into four groups and received specified doses of compound **2** via intraperitoneal injection (i.p.). (A) Tumor size was evaluated twice a week, and (B) body weight was recorded daily in the first week, followed by semiweekly measurements. Irinotecan was employed as the reference drug. Mean ± SD values represented tumor volume and body weight, with the percentage of tumor growth inhibition (%TGI) calculated. **p < 0.01 and ***p < 0.001 as compared with the control group.

An *in vivo* experiment was conducted to confirm the antitumor effect of **2**. HCT-116 cells were subcutaneously implanted in Balb/c nude mice, and compound **2** was administered at concentrations of 50 or 100 mg/kg to assess the antitumor capabilities. In the 50 mg/kg dose group, significant inhibition of tumor growth was observed, with a %TGI of 35.1% (Figure 8A). In the high-dose 100 mg/kg group, **2** led to increased tumor inhibition to 63.7% (Figure 8A). Based on body weight measurements, **2** did not cause evident toxicity in animals (Figure 8B). Notably, the tumor growth inhibition by the low dose of compound **2** was comparable to the clinically used irinotecan, with a %TGI of 36.9% (Figure 8A). In a nutshell, **2** not only effectively inhibited tumor growth but also achieved therapeutic efficacy similar to clinical drugs at lower concentrations. The results suggest that compound **2** exhibits potential for development as a therapeutic for treating CRC.

### CRC Patient-Derived Organoids

**Figure 9:**
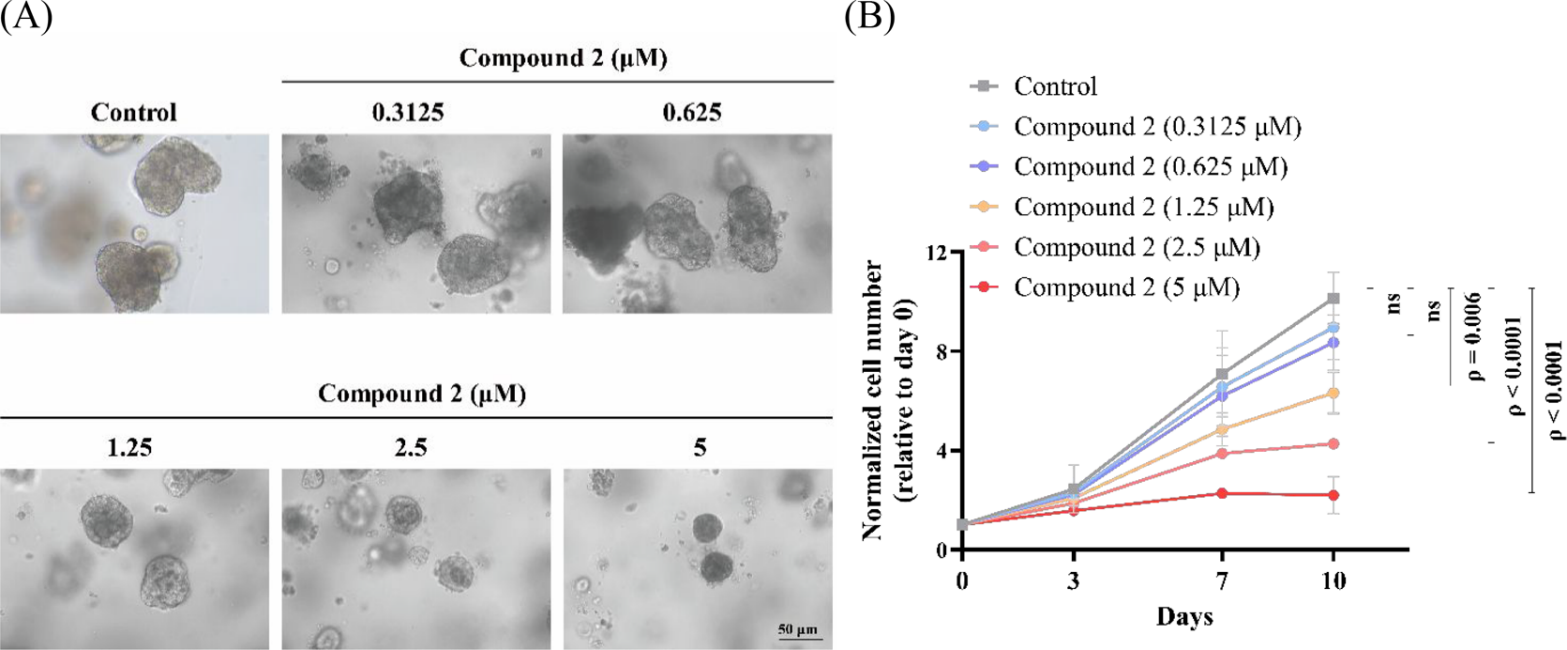
(A) Cell proliferation analysis of indicated treatment concentration in CRC PDOs. (B) A panel of PDOs is at the end point, and the line graphs show the proliferation rate of each treatment concentration. Data are shown as means ± S.E.M. (*n* = 3).

Patient-derived organoids (PDOs) have emerged as attractive models to recapitulate the architectural and genomic characteristics of the primary tumor and reflect and predict the responsiveness of the primary tumor. In testing and evaluation of compound **2** on human patient-derived CRC organoids, significant inhibition of CRC tissue growth was observed at concentrations of 1.25, 2.5, and 5 μM, with *p* = 0.006, *p* < 0.0001, and *p* < 0.0001, respectively (Figure 9A,B). Even on the 10th day, compound **2** at a concentration of 5 μM still exhibited notable suppression of cancer cell growth.

### Immunoblot Analysis of Drug-Treated CRC Patient-Derived Organoids

**Figure 10:**
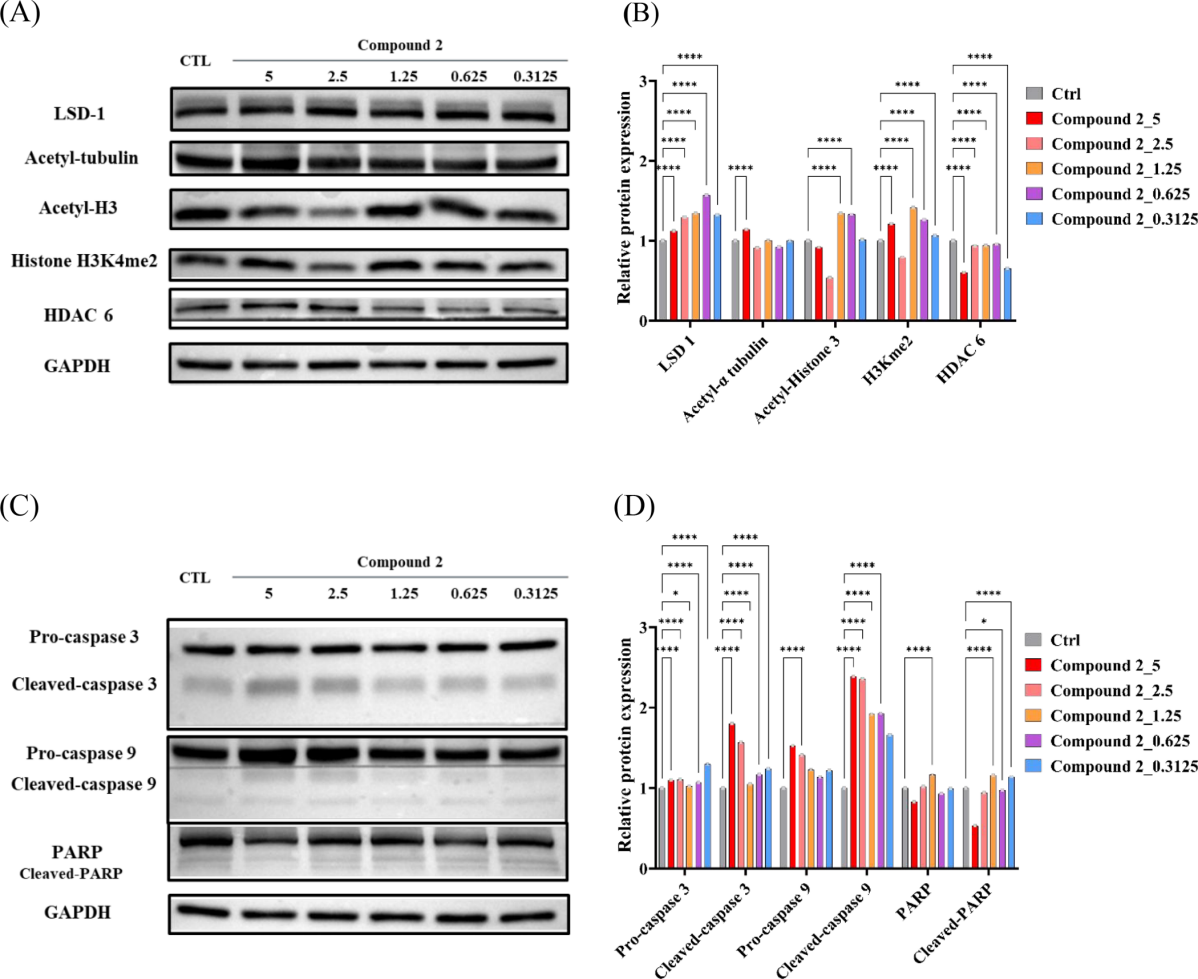
Western blot analysis and semiquantification. (A) Western blot analysis and (B) semiquantification of the HDAC inhibition and LSD1 relative biomarker proteins on lysates from CRC PDOs after 10 days of treatment with compound **2**. (C) Western blot analysis and (D) semiquantification of the apoptosis-related proteins on lysates from CRC PDOs after 10 days of treatment with compound **2**. Statistical significance in (B) and (D) is indicated by *p < 0.05, ***p < 0.001, and ****p < 0.0001 compared with the control group, as determined using one-way ANOVA.

To confirm the *in vivo* target engagement of compound **2**, CRC PDOs were treated with compound **2** for 10 days. To further investigate the drug response, we analyzed the results of Western blotting and quantified the levels of HDAC inhibition, LSD1 relative biomarkers, and apoptosis-related proteins in lysates from CRC PDOs. Compound **2** (1.25 μM) performed well, resulting in the degradation of HDAC6 by 39.5% compared to the control. Treatment with compound **2** led to the upregulation of acetyl-tubulin and acetyl-histone H3. Additionally, it induced the accumulation of H3K4me2 and apoptosis (*p* < 0.001) (Figure 10A,B). Treatment with compound **2** induced H3K4me2 accumulation and cell death, accompanied by the activation of caspases 3 and 9. The activated caspases subsequently broke down the cellular substrate PARP and induced the expression of cleaved-PARP protein, ultimately leading to cell death (*p* < 0.001) (Figure 10C,D).

### Immunohistochemistry (IHC) Staining of Drug-Treated CRC PDOs

**Figure 11:**
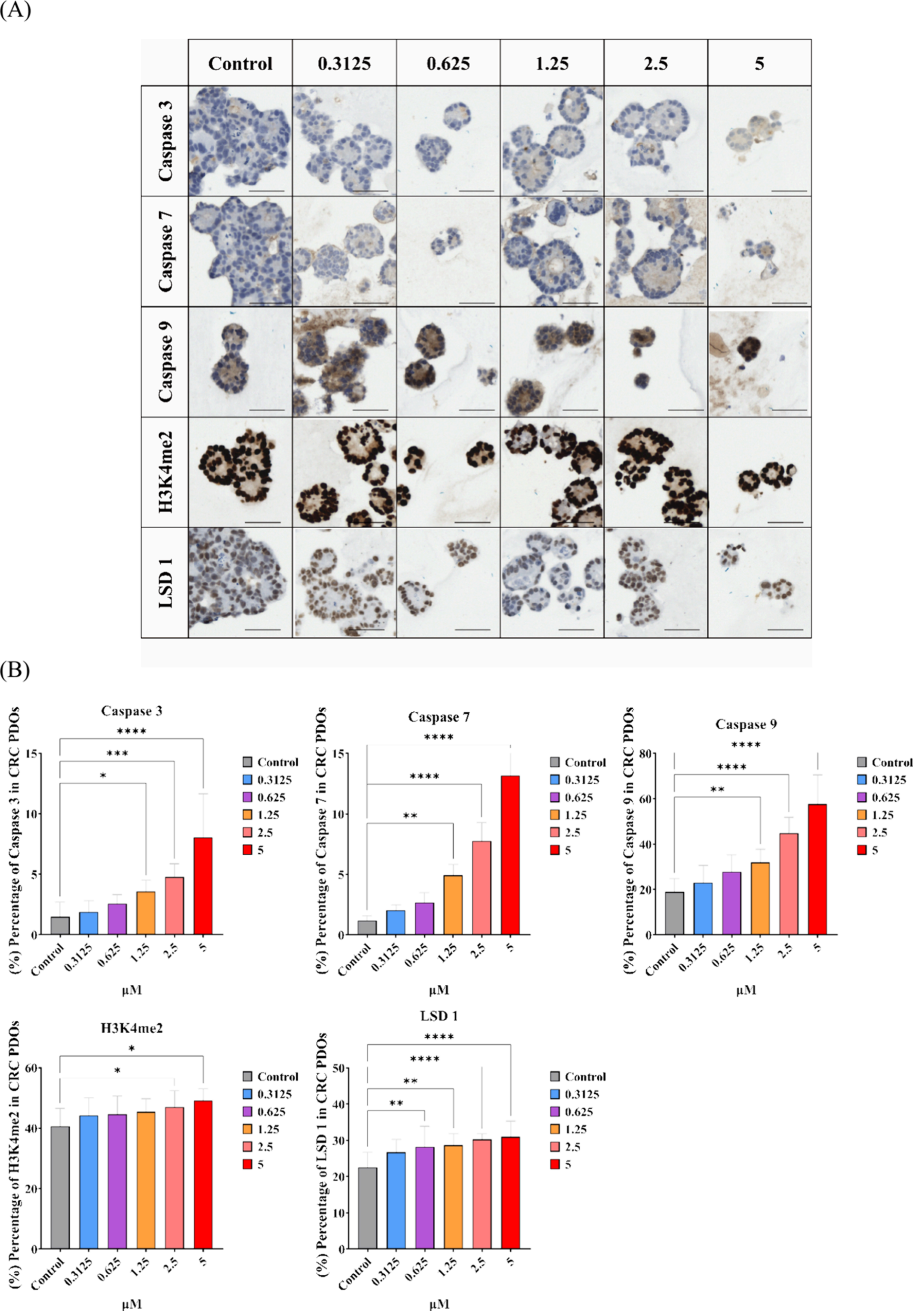
Immunohistochemistry (IHC) staining of compound 2-treated CRC PDOs. (A) IHC staining of apoptosis-related proteins, HDAC inhibition, and LSD1 relative biomarker proteins in the drug-treated CRC PDOs. (B) Quantification of staining intensities for the indicated markers was performed in 10 organoid samples. Scale bars, 100 μm.

PDOs have revolutionized cancer research by retaining histological and IHC features similar to the primary tumors from which they were derived. Therefore, PDOs serve as a valuable preclinical *ex vivo* model for predicting patient responses to chemotherapy regimens.^33,34^ To reveal the potential *in vivo* antitumor mechanism of CRC PDOs treated with compound **2**, we evaluated the IHC staining for the cleavage activation of caspases 3, 7, and 9, which are prominent markers of apoptosis, as well as the biomarkers histone H3K4me2 and LSD1 (Figure 11A). Levels of histone H3K4me2 and LSD1 were quantified to evaluate the *in vivo* target engagement of compound **2** in the CRC PDOs. As shown in Figure 11B, the IHC staining revealed that H3K4me2 and LSD1 were expressed at significantly higher levels in the groups treated with compound **2** at concentrations above 2.5 μM compared to the control group (*p* < 0.05 and *p* < 0.001, respectively). According to the IHC staining of caspases 3, 7, and 9 in drug-treated CRC PDOs, compound **2** at concentrations above 1.25 μM significantly increased the expression of these pro-apoptotic proteins compared to the control, demonstrating a dose-dependent effect (*p* < 0.05). The results showed that treatment with compound **2** led to the upregulation of LSD1, accumulation of H3K4me2, and activation of caspases 3, 7, and 9 in the *ex vivo* drug-treated CRC PDOs, explaining the excellent antitumor efficacy.

## Conclusions

This study involved the rational design of dual LSD1–HDAC inhibitors as promising anticancer interventions for CRC assembled via conjugation of key pharmacophores of LSD1 and HDAC inhibitors. The embarkment of this campaign stems from the reports confirming the overexpression of LSD1 and HDAC isoforms in CRC. Also, the precedential claims regarding the remarkable antitumor potential of dual LSD1–HDAC inhibitors in diverse malignancies strengthened our envisionment of extracting amplified anti-CRC effects via simultaneous inhibition of LSD1 and HDAC. Accordingly, a series of tranylcypromine-based hydroxamic acids was furnished and evaluated for anti-CRC effects. The evaluation of cell growth inhibitory effects of the synthesized adducts led us to pinpoint hydroxamic acid **2** endowed with striking antitumor activity against the colorectal cell lines.

Further evaluation underscored the ability of compound **2** to exert preferential inhibition of HDAC isoform and LSD1. The correlation of the results from cellular assay and enzymatic assays indicates the dual inhibition of LSD1–HDAC as the underlying mechanism for the magnificent cell growth inhibitory effects of compound **2**. The outcome of additional assays further supplemented the anti-CRC profile of **2** as it significantly reduced colony formation at a concentration of 0.625 μM after 3 days of treatment in HCT-116 cells and exerted cell cycle arrest at the G0/G1 phase, eventually leading to cell death. Moreover, the impact of compound **2** treatment on the protein levels of HDAC was observed by Western blot analysis, and the results demonstrated an upregulated expression of acetyl-tubulin and acetyl-histone H3 with escalating doses of compound **2**. Also, H3K4ME2 accumulation and cell death with activation of caspase 3/9 on treatment of compound **2** was observed (Western blot analysis). It is important to mention that the aforementioned increase in the levels of acetyl-tubulin, acetyl-histone, and histone H3K4 dimethylation (H3K4me2) aligns with the results of previous investigations on LSD1 and HDAC inhibitors. To evaluate whether the impressive *in vitro* anti-CRC activity of **2** is translated to *in vivo* antitumor activity, an *in vivo* study in Balb/c nude mice was conducted. Compound **2** (50 or 100 mg/kg dose) significantly inhibited tumor growth, with %TGI of 35.1 and 63.7%, respectively. The response to compound **2** in PDO cultures was consistent with the subcutaneous xenograft tumors in mice. In particular, our investigation focused on the screening results of compound **2** using PDOs derived from CRC patients. **2** exhibited significant inhibition of CRC tissue growth in human patient-derived CRC organoids in a dose-dependent manner, particularly evident over the concentration of 1.25 μM by the 10th day. Remarkably, compound **2** demonstrated significant *in vivo* target engagement in CRC PDOs, leading to a 39.5% degradation of HDAC6 and upregulation of acetyl-tubulin and acetyl-histone H3. The treatment induced H3K4me2 accumulation, activated caspases 3, 7, and 9, and increased cleaved-PARP protein expression, resulting in marked apoptosis. These results underscore the relevance of CRC organoid responses to compound **2** and their potential implications for clinical outcomes in CRC patients. Cumulatively, the results of this study present a tractable anti-CRC agent manifesting activity through dual modulation of LSD1 and HDAC.

## Experimental Section

### Chemistry

A Bruker DRX-500 spectrometer operating at 300 MHz was used for nuclear magnetic resonance (^1^H NMR and ^13^C NMR). A JEOL (JMS-700) electron impact (EI) mass spectrometer was used for HRMS. The Hitachi 2000 series HPLC system using a C18 column was used to assess the purity of the final compounds. All compounds are >95% pure by HPLC analysis.

#### Synthesis of Methyl 4-((((1*R*,2*S*)-2-Phenylcyclopropyl)amino)methyl)benzoate (**12**)

(1*R*,2*S*)-2-Phenylcyclopropan-1-amine hydrochloride (0.5 g, 2.9 mmol) was dissolved in DMF (5 mL). Potassium carbonate (0.610 g, 4.4 mmol) and methyl 4-bromobenzoate (0.665 mg, 3.09 mmol) were added to the reaction mixture. The reaction mixture was kept in a stirred condition for 3 h at 60 °C. The completion of the reaction was monitored by TLC. The reaction mixture was quenched with water and then extracted with ethyl acetate (50 mL × 3). The ethyl acetate layers were combined, dried over anhydrous MgSO_4_, and concentrated under reduced pressure. Silica gel chromatography (EtOAc:*n*-hexane = 1:4) was used for the purification of the residue that yielded compound **1** in 75% yield; ^1^H NMR (300 MHz, DMSO): 7.97 (d, *J* = 8.4 Hz, 2H), 7.46 (d, *J* = 7.5 Hz, 2H), 7.12–7.20 (m, 3H), 7.00 (m, 2H), 3.95 (s, 3H), 2.36 (m, 1H), 1.92 (m, 1H), 1.12 (m, 1H), 1.03 (m, 1H).

#### Synthesis of 4-((((1*R*,2*S*)-2-Phenylcyclopropyl)amino)methyl)benzoic Acid (**13**)

Lithium hydroxide [(1 M (aq) (5 mL)] was added to the solution of compound **12** (0.4 g, 1.4 mmol) in dioxane (5 mL). The reaction mixture was stirred for 2 h. Upon completion of the reaction, as monitored by TLC, water was added to the reaction mixture. The reaction mixture was then acidified with 3N HCL and then extracted with ethyl acetate (20 mL × 3). The combined organic layers were concentrated under reduced pressure to yield the acid **13** in 92% yield. The carboxylic acid obtained was used for further reactions without purification.

#### Synthesis of *N*-Hydroxy-4-((((1*R*,2*S*)-2-phenylcyclopropyl)amino)methyl)benzamide (**1**)

Compound **13** (0.2 g, 0.7 mmol) was dissolved in DMF (5 mL). To the solution, EDC·HCl (0.286 g, 1.44 mmol), HOBt (0.189 mg, 1.44 mmol), and DIPEA (0.365 mL, 2.98 mmol) in DMF (5 mL) were added and the reaction mixture was stirred at room temperature for 1 h before adding NH_2_OTHP (0.09 g, 0.77 mmol). After 4 h, water was added to the reaction mixture and extraction was done with ethyl acetate (20 mL × 3). The combined ethyl acetate layers were concentrated under reduced pressure and the residue was purified by silica gel chromatography (EtOAc). The obtained residue (300 mg) was dissolved in CH_3_OH (5 mL), and 10% TFA_(aq)_ (5 mL) was added to the solution. The reaction mixture was then stirred for 3 h and concentrated under reduced pressure to get a white precipitate (**1**) in 51% yield; HPLC purity: 98.97%; ^1^H NMR (300 MHz, CD_3_OD): 7.90 (d, *J* = 8.4 Hz, 2H), 7.48 (d, *J* = 8.4 Hz, 2H), 7.18–7.30 (m, 5H), 3.94 (s, 2H), 2.65–2.78 (m, 2H), 1.90–1.95 (m, 2H). ^13^C NMR (150 MHz, CD_3_OD-d_4_) δ 26.67, 28.63, 32.49, 51.11, 125.83, 127.83, 127.96, 128.14, 129.05, 133.86, 137.90, 140.69, 170.19.

#### Synthesis of 3-Bromo-*N*-((1*R*,2*S*)-2-phenylcyclopropyl)benzenesulfonamide (**14**)

Starting material **1** (1 g, 5.89 mmol) was dissolved in DCM (15 mL). 3-Bromobenzenesulfonyl chloride (1.5 g, 5.89 mmol) and triethyl amine (1.64 mL, 11.78 mmol) were added to the solution, and the reaction mixture was stirred for 3 h. After 3 h, water was added to quench the reaction mixture. Extraction was done with ethyl acetate (30 mL × 3) and the combined ethyl acetate layer was concentrated under reduced pressure. The residue obtained was purified by silica gel chromatography (hexane:ethyl acetate = 3:1) that yielded compound **14** in 87% yield. ^1^H NMR (300 MHz, CDCl_3_): 7.77 (m, 1H), 7.71 (m, 1H), 7.22–7.41 (m, 5H), 6.82 (m, 2H), 2.34 (m, 1H), 1.99 (m, 1H), 1.15–1.21 (m, 2H).

#### Synthesis of 4-Bromo-*N*-((1*R*,2*S*)-2-phenylcyclopropyl)benzenesulfonamide (**15**)

Compound **15** was furnished using compound **1** and 4-bromobenzene sulfonyl chloride as per the methodology described for the synthesis of intermediate **14** in 82% yield. ^1^H NMR (300 MHz, CDCl_3_): 7.72 (d, *J* = 8.4 Hz, 2H), 7.69 (d, *J* = 8.4 Hz, 2H), 6.98–7.08 (m, 5H), 2.22 (m, 1H), 1.91 (m, 1H), 1.09–1.18 (m, 2H).

#### Synthesis of Methyl (*E*)-3-(3-(*N*-((1*R*,2*S*)-2-Phenylcyclopropyl)sulfamoyl)phenyl)acrylate (**16**)

A mixture of compound **14** (700 g, 1.98 mmol), palladium(II) acetate (0.178 g, 0.79 mmol), triphenyl phosphine (103 mg, 0.396 mmol), triethylamine (0.711 mL, 5.10 mmol), and methyl acrylate (0.205 mL, 2.26 mmol) in DMF (10 mL) was stirred at 100 °C for 6 h. Water was used to quench the reaction, and extraction was done with ethyl acetate (50 mL × 3). Ethyl acetate layers were combined and concentrated under reduced pressure to yield a residue that was subjected to purification by silica gel chromatography (EtOAc:*n*-hexane = 2:3) to get compound **24** in 49% yield; ^1^H NMR (300 MHz, CD_3_OD): 7.87 (m, 1H), 7.79 (d, *J* = 7.8 Hz, 2H), 7.42–7.49 (m, 2H), 7.08–7.21 (m, 3H), 6.86 (d, *J* = 7.5 Hz, 2H), 6.42 (d, *J* = 15.9 Hz, 1H), 3.81 (s, 3H) 2.31 (bs, 1H), 1.94 (bs, 1H), 1.07–1.15 (m, 2H).

#### Synthesis of Methyl (*E*)-3-(4-(*N*-((1*R*,2*S*)-2-Phenylcyclopropyl)sulfamoyl)phenyl)acrylate (**17**)

Compound **17** was furnished using compound **15** as per the methodology described for the synthesis of intermediate **16** in 52% yield. ^1^H NMR (300 MHz, CD_3_OD): 7.78 (d, *J* = 8.1 Hz, 2H), 7.65 (d, *J* = 8.1 Hz, 2H), 7.61 (d, *J* = 15.6 Hz, 1H), 7.08–7.17 (m, 3H), 6.91 **-** 6.93 (m, 2H), 6.58 (d, *J* = 15.6 Hz, 1H), 3.79 (s, 3H), 2.28 (m, 1H), 1.94 (bs, 1H), 1.11–1.15 (m, 2H).

#### Synthesis of (*E*)-3-(3-(*N*-((1*R*,2*S*)-2-Phenylcyclopropyl)sulfamoyl)phenyl)acrylic Acid (**18**)

Acid **18** was generated in 93% yield in a manner similar to that described for the synthesis of **13.** Intermediate **18** was utilized for further reactions without purification.

#### Synthesis of (*E*)-3-(4-(*N*-((1*R*,2*S*)-2-Phenylcyclopropyl)sulfamoyl)phenyl)acrylic Acid (**19**)

Acid **19** was generated in 93% yield in a manner similar to that described for the synthesis of **13**. Intermediate **19** was utilized for further reactions without purification.

#### Synthesis of (*E*)-*N*-Hydroxy-3-(3-(*N*-((1*R*,2*S*)-2-phenylcyclopropyl)sulfamoyl)phenyl)acrylamide (**2**)

Hydroxamic acid **2** was obtained in 66% yield in a manner similar to that described for the synthesis of compound **1**. HPLC purity: 100%; ^1^H NMR (300 MHz, CD_3_OD): 7.95 (m, 1H), 7.82 (d, *J* = 6.9 Hz, 2H), 7.51–7.62 (m, 2H), 7.11–7.24 (m, 3H), 6.91 (d, *J* = 7.5 Hz, 2H), 6.46 (d, *J* = 15.6 Hz, 1H), 2.34 (bs, 1H), 1.95 (bs, 1H), 1.11–1.19 (m, 2H).^13^C NMR (150 MHz, CD_3_OD-d_4_) δ 13.74, 23.56, 33.88, 119.47, 125.42, 125.68, 125.91, 127.62, 127.97, 129.55, 131.39, 135.96, 138.11, 140.11, 140.89, 164.10; HRMS (ESI) for C_18_H_19_N_2_O_4_S [M + H]^+^: calcd, 359.1066; found, 359.1066; [α]_D_^25^ = −7.20° (c 0.5, MeOH).

#### (*E*)-*N*-Hydroxy-3-(4-(*N*-((1*R*,2*S*)-2-phenylcyclopropyl)sulfamoyl)phenyl)acrylamide (**3**)

Hydroxamic acid **3** was obtained in 41% yield in a manner similar to that described for the synthesis of compound 1. HPLC purity: 95.46%; ^1^H NMR (300 MHz, CD_3_OD): 7.83 (d, *J* = 8.1 Hz, 2H), 7.70 (d, *J* = 8.4 Hz, 2H), 7.60 (d, *J* = 15.9 Hz, 1H), 7.12–7.25 (m, 3H), 6.93–6.96 (m, 2H), 6.60 (d, *J* = 15.9 Hz, 1H), 2.33 (m, 1H), 1.96 (m, 1H), 1.09–1.18 (m, 2H). ^13^C NMR (150 MHz, DMSO-d_6_) δ 14.77, 23.74, 34.60, 126.18, 126.20, 126.26, 127.80, 128.36, 128.61, 140.68, 162.78. HRMS (ESI) for C_18_H_19_N_2_O_4_S [M + H]^+^: calcd, 359.1066; found, 359.1066.

#### Synthesis of Methyl 7-Oxo-7-(((1*R*,2*S*)-2-phenylcyclopropyl)amino)heptanoate (**20**)

Compound **1** (0.5 g, 2.94 mmol) was dissolved in DMF (5 mL), and EDC·HCl (1.12 g, 5.89 mmol), HOBt (0.780 g, 5.78 mmol), and DIPEA (1.5 mL, 8.67 mmol) were added to the solution. The reaction mixture was stirred at room temperature for 1 h before adding 7-methoxy-7-oxoheptanoic acid (0.604 g, 3.46 mmol). After 4 h, water was added to the reaction mixture and extraction was done with ethyl acetate (20 mL × 3). The combined ethyl acetate layers were concentrated under reduced pressure, and the residue was purified by silica gel chromatography (EtOAc) to give compound **20** in 74% yield; HPLC purity: 100%; ^1^H NMR (300 MHz, CD_3_OD): 7.21–7.27 (m, 5H), 3.51 (s, 3H), 2.89 (m, 1H), 2.12–2.16 (m, 5H), 1.29–1.53 (m, 8H).

#### Synthesis of Methyl 8-Oxo-8-(((1*R*,2*S*)-2-phenylcyclopropyl)amino)octanoate (**21**)

Intermediate **21** was synthesized in 72% yield via amide bond formation between compound **1** and monomethyl suberate employing the carbodiimide-based methodology, as described for the synthesis of compound **20**. ^1^H NMR (300 MHz, CD_3_OD): 7.19–7.21 (m, 5H), 3.53 (s, 3H), 2.82 (m, 1H), 2.14–2.21 (m, 5H), 1.66 (bs, 4H), 1.12–1.23 (m, 6H).

#### Synthesis of Methyl 9-Oxo-9-(((1*R*,2*S*)-2-phenylcyclopropyl)amino)nonanoate (**22**)

Intermediate **22** was synthesized in 68% yield via amide bond formation between compound **1** and 9-methoxy-9-oxononanoic acid employing the carbodiimide-based methodology, as described for the synthesis of compound **20**. ^1^H NMR (300 MHz, CD_3_OD): 7.21–7.36 (m, 5H), 3.61 (s, 3H), 2.81 (m, 1H), 2.11–2.13 (m, 5H), 1.71 (bs, 4H), 1.28 (bs, 6H), 1.09–1.13 (m, 2H).

#### Synthesis of Methyl 10-Oxo-10-(((1*R*,2*S*)-2-phenylcyclopropyl)amino)decanoate (**23**)

Intermediate **23** was synthesized in 69% yield via amide bond formation between compound **1** and 10-methoxy-10-oxodecanoic acid, employing the carbodiimide-based methodology as described for the synthesis of compound **20**. ^1^H NMR (300 MHz, CD_3_OD): 7.11–7.21 (m, 5H), 3.57 (s, 3H), 2.79 (m, 1H), 2.18–2.24 (m, 5H), 1.63 (bs, 4H), 1.17–1.32 (m, 8H).

#### Synthesis of N1-Hydroxy-N7-((1*R*,2*S*)-2-phenylcyclopropyl)heptanediamide (**4**)

Hydroxamic acid **4** was synthesized in 62% yield in a manner similar to that described for the synthesis of compound **1**. HPLC purity: 100%; ^1^H NMR (300 MHz, CD_3_OD): 7.16–7.31 (m, 5H), 2.83 (m, 1H), 2.08–2.16 (m, 5H), 1.53–1.58 (m, 4H), 1.29–1.35 (m, 4H). ^13^C NMR (150 MHz, CD_3_OD-d_4_) δ 14.65, 18.97, 24.91, 25.15, 28.08, 32.11, 35.49, 42.02, 46.32, 125.87, 127.85, 127.88, 138.68, 171.42, 173.75; HRMS (ESI) for C_16_H_23_N_2_O_3_ [M + H]^+^: calcd, 291.1709; found, 291.1708.

#### Synthesis of N1-Hydroxy-N8-((1*R*,2*S*)-2-phenylcyclopropyl)octanediamide (**5**)

Hydroxamic acid **5** was synthesized in 55% yield in a manner similar to that described for the synthesis of compound **1.** HPLC purity: 100%; ^1^H NMR (300 MHz, CD_3_OD): 7.14–7.27 (m, 5H), 2.87 (m, 1H), 2.04–2.23 (m, 5H), 1.64 (bs, 4H), 1.18–1.23 (m, 4H), 1.13–1.15 (m, 2H). ^13^C NMR (150 MHz, CD_3_OD-d_4_) δ 14.67, 23.75, 25.13, 25.30, 28.33, 28.41, 31.81, 35.35, 42.02, 125.53, 125.80, 127.85, 127.89, 128.88, 140.87, 171.52, 176.03; HRMS (ESI) for C_17_H_25_N_2_O_3_ [M + H]^+^: calcd, 305.1865; found, 305.1866.

#### Synthesis of N1-Hydroxy-N9-((1*R*,2*S*)-2-phenylcyclopropyl)nonanediamide (**6**)

Hydroxamic acid **6** was synthesized in 63% yield in a manner similar to that described for the synthesis of compound **1.** HPLC purity: 99.69%; ^1^H NMR (300 MHz, CD_3_OD): 7.14–7.30 (m, 5H), 2.85 (m, 1H), 2.02–2.22 (m, 5H), 1.66 (bs, 4H), 1.31 (bs, 6H), 1.13–1.31 (m, 2H). ^13^C NMR (150 MHz, CD_3_OD-d_4_) δ 14.66, 23.75, 25.23, 25.40, 28.50, 28.64, 31.81, 32.31, 35.42, 42.02, 46.31, 125.53, 125.80, 127.89, 128.77, 140.86, 171.56, 176.09; HRMS (ESI) for C_18_H_27_N_2_O_3_ [M + H]^+^: calcd, 319.2022; found, 319.2020.

#### Synthesis of N1-Hydroxy-N10-((1*R*,2*S*)-2-phenylcyclopropyl)decanediamide (**7**)

Hydroxamic acid **7** was synthesized in 51% yield in a manner similar to that described for the synthesis of compound **1**. HPLC purity: 99.74%; ^1^H NMR (300 MHz, CD_3_OD): 7.17–7.29 (m, 5H), 2.75 (m, 1H), 2.08–2.14 (m, 5H), 1.61–1.65 (m, 4H), 1.21–1.36 (m, 8H). ^13^C NMR (150 MHz, CD_3_OD-d_4_) δ 19.01, 25.29, 25.58, 28.63, 28.65, 28.71, 28.79, 32.34, 35.77, 42.01, 46.30, 125.53, 125.80, 127.84, 128.88, 138.68, 171.58, 173.99; HRMS (ESI) for C_19_H_29_N_2_O_3_ [M + H]^+^: calcd, 333.2178; found, 333.2178.

#### Synthesis of (1*R*,2*S*)-*N*-(4-Nitrobenzyl)-2-phenyl Cyclopropane-1-amine (**24**)

Compound **24** was synthesized via benzylation of compound **1** with 4-nitrobenzyl bromide in a manner similar to that described for the synthesis of **12**. ^1^H NMR (300 MHz, CD_3_OD): 8.21 (d, *J* = 8.1 Hz, 2H), 7.67 (d, *J* = 8.1 Hz, 2H), 7.00–7.12 (m, 5H), 3.92 (s, 2H), 2.39 (m, 1H), 1.98 (m, 1H), 1.27 (m, 1H), 1.13 (m, 1H).

#### Synthesis of 4-((((1*R*,2*S*)-2-Phenylcyclopropyl)amino)methyl)aniline (**25**)

Iron powder (1.04 g, 18.6 mmol) and ammonium chloride (0.397 g, 7.44 mmol) were added to the solution of compound **24** (1g, 3.7 mmol) in ethanol (45 mL) and water (5 mL). The reaction mixture was refluxed for 5 h, and then workup was done. The resulting residue (80% yield) was used for further reactions with purification.

#### Synthesis of Methyl 8-Oxo-8-((4-((((1*R*,2*S*)-2-phenylcyclopropyl)amino)methyl)phenyl)amino)octanoate (**26**)

Intermediate **26** was synthesized in 62% yield via amide bond formation between compound **25** and monomethyl suberate employing the carbodiimide-based methodology, as described for the synthesis of compound **20**.^1^H NMR (300 MHz, CD_3_OD): 7.48 (d, *J* = 8.4 Hz, 2H), 7.11–7.27 (m, 5H), 7.08 (d, *J* = 7.2 Hz, 2H), 3.88 (s, 3H), 3.78 (s, 2H), 2.21–2.37 (m, 5H), 1.65–1.94 (m, 5H), 1.48 (bs, 4H), 1.13 (m, 1H), 1.08 (m,1H).

#### Synthesis of 8-Oxo-8-((4-((((1*R*,2*S*)-2-phenylcyclopropyl)amino)methyl)phenyl)amino)octanoic Acid (**27**)

Acid **27** was generated in 89% yield in a manner similar to that described for the synthesis of **13**. Intermediate **27** was utilized for further reactions without purification.

#### Synthesis of N1-Hydroxy-N8-(4-((((1*R*,2*S*) phenylcyclopropyl)amino)methyl)phenyl)octanediamide (**8**)

Hydroxamic acid **8** was obtained in 26% yield in a manner similar to that described for the synthesis of compound **1**. HPLC purity: 98.76%; ^1^H NMR (300 MHz, CD_3_OD): 7.50 (d, *J* = 8.4 Hz, 2H), 7.20–7.29 (m, 4H), 7.14 (m, 1H), 7.00 (d, *J* = 7.2 Hz, 2H), 3.83 (s, 2H), 2.39 (t, *J* = 7.2 Hz, 2H), 2.32 (m, 1H), 2.14 (t, *J* = 7.2 Hz, 2H), 1.94 (m, 1H), 1.63–1.172 (m, 4H), 1.43 (bs, 4H), 1.03 (m, 1H), 0.99 (m,1H). ^13^C NMR (150 MHz, CD_3_OD-d_4_) δ 14.56, 23.25, 23.27, 24.27, 33.30, 34.37, 50.50, 53.81, 53.82, 59.43, 61.26, 125.77, 125.92, 127.99, 130.05, 131.20, 132.34, 133.53, 140.18, 167.49; HRMS (ESI) for C_24_H_32_N_3_O_3_ [M + H]^+^: calcd, 410.2444; found, 410.2447.

#### Synthesis of *tert*-Butyl 4-((((1*R*,2*S*)-2-Phenylcyclopropyl)amino)methyl)piperidine-1-carboxylate (**28**)

Compound **1** (1 g, 5.89 mmol) was dissolved in methanol, and aq. NH_4_OH (5 mL) was added to the solution. The reaction mixture was then stirred for 2 h at room temperature. Water was added to it, and extraction was done with ethyl acetate (50 mL × 3). The combined ethyl acetate layer was dried over MgSO_4_ and concentrated under reduced pressure to get a residue (a nonionic form of tranylcypromine, 0.7 g). The residue (0.7 g, 5.2 mmol) was then dissolved in DCM (5 mL), and *tert*-butyl 4-formylpiperidine-1-carboxylate (1.12 g, 5.2 mmol) was added to the solution. Acetic acid (0.1 mL) was added to the reaction mixture, and the reaction mixture was stirred for 2 h at room temperature before adding sodium triacetoxy borohydride (2.2 g, 10.51 mmol). After stirring for 12 h, water was added and DCM (50 mL × 3) was used for the extraction. The combined DCM layer was concentrated under reduced pressure, and the residue obtained was purified by silica gel chromatography (DCM:CH_3_OH = 9.5:0.5) that yielded compound **28** in 41% yield. ^1^H NMR (300 MHz, CD_3_OD): 7.13–7.29 (m, 5H), 3.44 (s, 2H), 3.29–3.31 (m, 3H), 3.05 (m, 2H), 2.75 (m, 1H), 2.18–2.28 (m, 2H), 1.86–2.14 (m, 3H), 1.47 (s, 9H), 1.26–1.31 (m, 2H).

#### Synthesis of *tert*-Butyl 4-((2,2,2-Trifluoro-*N*-((1*R*,2*S*)-2-phenylcyclopropyl)acetamido)methyl)piperidine-1-carboxylate (**29**)

Compound **28** (0.4 g, 1.2 mmol) was dissolved in DCM (5 mL), and trifluoroacetic anhydride (1 mL) was added to the solution. The reaction mixture was stirred for 3 h at room temperature. Water was added to the reaction mixture, and extraction was done with DCM (20 mL × 3). The combined DCM layer was concentrated under reduced pressure, and the residue obtained (500 mg) was used for further reactions without purification.

#### Synthesis of 2,2,2-Trifluoro-*N*-((1*R*,2*S*)-2-phenylcyclopropyl)-*N*-(piperidin-4-ylmethyl)acetamide (**30**)

Compound **29** (300 mg, 0.7 mmol) was dissolved in DCM (2 mL), and trifluoroacetic acid (2 mL) was added to it. The reaction mixture was stirred for 3 h. After 3 h, water was added to the reaction mixture and sodium bicarbonate (10% aq) was added to basify. Extraction was done with ethyl acetate (30 mL × 3). The combined ethyl acetate layers were concentrated under reduced pressure, and the residue obtained was subjected to purification using silica gel chromatography (DCM:methanol = 9.5::0.5) to get **34** in 79% yield. ^1^H NMR (300 MHz, CD_3_OD): 7.23–7.34 (m, 5H), 3.57 (s, 2H), 3.25–3.39 (m, 3H), 3.09 (m, 2H), 2.79 (bs, 1H), 2.25–2.37 (m, 2H), 1.84–2.09 (m, 3H), 1.27–1.38 (m, 2H).

#### Synthesis of Methyl 4-((4-((2,2,2-Trifluoro-*N*-((1*R*,2*S*)-2-phenylcyclopropyl)acetamido)methyl)piperidin-1-yl)methyl)benzoate (**31**)

Benzylation of compound **30** was carried out using methyl 4-(bromomethyl)benzoate in a manner similar to that described for the synthesis of **12**. ^1^H NMR (300 MHz, CD_3_OD):7.99 (d, *J* = 7.5 Hz, 2H), 7.47 (d, *J* = 8.1 Hz, 2H), 7.14–7.45 (m, 5H), 4.62 (s, 2H), 3.84 (s, 3H), 3.60 (s, 2H), 3.21–3.32 (m, 3H), 3.19 (m, 2H), 2.89 (bs, 1H), 2.31–2.38 (m, 2H), 1.88–2.12 (m, 3H), 1.33–1.38 (m, 2H).

#### Synthesis of 4-((4-((((1*R*,2*S*)-2-Phenylcyclopropyl)amino)methyl)piperidin-1-yl)methyl)benzoic Acid (**32**)

Compound **31** (0.2 g, 0.42 mmol) was dissolved in methanol (1 mL), and 50 aq. NaOH (1 mL) was added to it. The reaction mixture was kept in stirred conditions for 5 h at room temperature. Upon completion of the reaction, as monitored by TLC, water was added to the reaction mixture. The reaction mixture was then acidified with 3 N HCl and then extracted with ethyl acetate (20 mL × 3). The combined organic layers were concentrated under reduced pressure to yield acid **32** in 83% yield. ^1^H NMR (300 MHz, CD_3_OD): 8.12 (d, *J* = 8.1 Hz, 2H), 7.67 (d, *J* = 8.1 Hz, 2H), 7.16–7.34 (m, 5H), 4.40 (s, 2H), 3.59 (s, 2H), 3.11–3.18 (m, 3H), 2.73 (m, 1H), 1.81–2.14 (m, 7H), 1.21–1.29 (m, 2H).

#### Synthesis of *N*-Hydroxy-4-((4-((((1*R*,2*S*)-2-phenylcyclopropyl)amino)methyl)piperidin-1-yl)methyl)benzamide (**9**)

Hydroxamic acid **9** was obtained in 32% yield in a manner similar to that described for the synthesis of compound **1**. HPLC purity: 100%; ^1^H NMR (300 MHz, CD_3_OD): 7.87 (d, *J* = 7.8 Hz, 2H); 7.63 (d, *J* = 7.8 Hz, 2H), 7.29–7.34 (m, 2H), 7.15–7.24 (m, 3H), 4.37 (s, 2H), 3.54 (s, 2H), 2.97–3.01 (m, 3H), 2.33 (m, 1H), 1.97–2.07 (m, 3H), 1.45–1.54 (m, 6H). ^13^C NMR (150 MHz, CD_3_OD-d_4_) δ 17.29, 21.50, 27.02, 32.52, 40.62, 49.36, 52.19, 52.22, 59.64, 125.25, 126.03, 127.54, 128.23, 131.24, 132.46, 133.75, 139.68, 165.58, 174.93; HRMS (ESI) for C_23_H_28_N_3_O_2_ [M - H]^+^: calcd, 378.2182; found, 378.2179.

#### Synthesis of *tert*-Butyl 4-(*N*-((1*R*,2*S*)-2-Phenylcyclopropyl)sulfamoyl)piperidine-1-carboxylate (**33**)

Starting material **1** (0.7 g, 4.12 mmol) was dissolved in DCM (10 mL). *tert*-Butyl 4-(chlorosulfonyl)piperidine-1-carboxylate (1.1 g, 4.12 mmol) and triethyl amine (1.15 mL, 8.26 mmol) were added to the solution, and the reaction mixture was stirred for 3 h at room temperature. Water was added to quench the reaction and DCM was used for the extraction (20 mL × 3). The combined organic layer was concentrated under reduced pressure, and the residue obtained was dissolved in DCM, Trifluoroacetic acid (neat) was added to the reaction mixture. The reaction mixture was stirred for 3 h. After 3 h, water was added to the reaction mixture and sodium bicarbonate (10% aq) was added to basify. Extraction was done with ethyl acetate (30 mL × 3). The combined ethyl acetate layers were concentrated under reduced pressure, and the residue obtained was subjected to purification using silica gel chromatography (DCM:methanol = 9.5:0.5) to get **34** in 79% yield. ^1^H NMR (300 MHz, CD_3_OD): 7.13–7.29 (m, 5H), 3.33–3.51 (m, 3H), 3.00 (m, 2H), 2.71 (bs, 1H), 2.21–2.34 (m, 2H), 1.98–2.04 (m, 3H), 1.30–1.34 (m, 2H).

#### Synthesis of Methyl 4-((4-(*N*-((1*R*,2*S*)-2-Phenylcyclopropyl)sulfamoyl)piperidin-1-yl)methyl)benzoate (**34**)

Compound **35** was synthesized in 74% yield in a manner similar to that described for the synthesis of **12**. ^1^H NMR (300 MHz, CD_3_OD): 8.01 (d, *J* = 8.4 Hz, 2H), 7.48 (d, *J* = 8.1 Hz, 2H), 7.12–7.29 (m, 5H), 3.93 (s, 3H), 3.62 (s, 2H), 3.00–3.12 (m, 3H), 2.69 (m, 1H), 1.86–2.20 (m, 7H), 1.20–1.27 (m, 2H).

#### Synthesis of 4-((4-(*N*-((1*R*,2*S*)-2-Phenylcyclopropyl)sulfamoyl)piperidin-1-yl)methyl)benzoic Acid (**35**)

Compound **36** was synthesized in 74% yield in a manner similar to that described for the synthesis of **13** and was used for further reactions without purification.

#### Synthesis of *N*-Hydroxy-4-((4-(*N*-((1*R*,2*S*)-2-phenylcyclopropyl)sulfamoyl)piperidin-1-yl)methyl)benzamide (**10**)

Compound **36** was synthesized in 46% yield in a manner similar to that described for the synthesis of **1**. HPLC purity: 99.44%; ^1^H NMR (300 MHz, CD_3_OD): 7.88 (d, *J* = 7.2 Hz, 2H), 7.63 (d, *J* = 6.9 Hz, 2H), 7.13–7.29 (m, 5H), 4.42 (s, 2H), 3.63 (bs, 2H), 3.45 (bs, 1H), 3.16 (bs, 2H), 2.70 (bs, 1H), 2.10–2.26 (m, 5H), 1.24–1.35 (m, 2H). ^13^C NMR (150 MHz, CD_3_OD-d_4_) δ 22.30, 25.49, 26.67, 28.63, 29.39, 32.49, 51.11, 125.83, 127.83, 127.96, 128.14, 129.05, 133.86, 137.90, 140.69, 170.19; HRMS (ESI) for C_22_H_28_N_3_O_4_S [M + H]^+^: calcd, 430.1811, found, 430.1799.

### Biology

#### Cell Culture

The human colorectal HCT-116 cell line was cultured in McCoy’s 5A medium (Corning, New York, USA) supplemented 10% (*v*/v) fetal bovine serum (FBS) (Corning, New York, USA) and 1% (v/v) penicillin–streptomycin–amphotericin B (PSA). Cells were grown in an incubator (37 °C, 5% CO_2_).

#### Chemicals and Antibodies

Cell Counting Kit-8 was provided by DOJINDO Laboratories (Kumamoto, Kyushu, Japan). 0.05% crystal violet staining solution and protease inhibitor cocktail were obtained from APExBIO (Boston, Massachusetts, USA). The Cell Cycle Phase Determination kit was purchased from Cayman Chemical (Ann Arbor, Michigan, USA). Radioimmunoprecipitation assay (RIPA) buffer was purchased from BIOMAN Scientific (Taipei, Taiwan). Phosphatase Inhibitor Cocktail 3, Dipase-II, and formaldehyde were obtained from Sigma-Aldrich (St. Louis, Missouri, USA). Antiacetyl-α-tubulin (Lys40), antiacetyl-Histone H3 (Lys9), anti-LSD1, anti-Caspase-3, and anti-Caspase-9 were obtained from Cell Signaling Technology (Danvers, Massachusetts, USA). Anti-Histone H3K4me2 (Dimethyl Lys4), anti-PARP, anti-GAPDH, horseradish peroxidase (HRP)-conjugated goat antimouse (1:10,000 dilution), and HRP-conjugated goat antirabbit were provided by GeneTex Inc. (Irvine, California, USA). Anti-Caspase-7 was produced from Novus Biologicals (Centennial, Colorado, USA). Antibeta actin was obtained from Santa Cruz Biotechnology (Dallas, Texas, USA). HistoGel was provided by Thermo Fisher Scientific (Waltham, Massachusetts, USA). Tissue-Clear Xylene Substitute and Glas Tissue-Mount were purchased by Sakura Finetek (Tokyo, Japan). Hematoxylin and eosin were obtained from Histolab Products AB (Askim, Sweden). Antibody diluent was provided by Agilent Technologies (Santa Clara, California, USA). Vector NovaRED Substrate Kit, Peroxidase (HRP) was obtained from Vector Laboratories (Newark, California, USA).

#### Cell Proliferation Assay

A Cell Counting Kit-8 (CCK-8, Dojindo, Kumamoto, Kyushu, Japan) assay was used to observe the effect of MPT1A drugs on cell proliferation.^35^ Before treatment with drugs, HCT-116 cells (1 × 10^4^ cells per well) were seeded into 96-well plates for 24 h, and then the cells were treated with 200 μL per well of full medium (10% FBS) containing different concentrations of test compounds, vorinostat (SAHA) or tranylcypromine (Tra), for 48 h. After treatment with the drug, 20 μL of detection reagent was added to each well and then it was incubated at 37 °C for 2 h. The formation of formazan was measured by the absorbance at 450 nm. Cell viability was expressed as the percentage of surviving cells in drug-treated versus DMSO-treated control cells (which was considered as 100% viability).

#### Colony Formation Assay

Colony formation assay was performed as per the protocol described earlier.^36^

#### Cell Cycle Analysis

HCT-116 cells were seeded at a density of 1 × 10^6^ cells per well in six-well plates and allowed to attach overnight. The culture medium was changed to serum-free to facilitate cycle synchronization prior to treatment with indicated concentrations of MPT1A drugs, SAHA or tranylcypromine. After 24 or 48 h, cells were harvested with 0.25% trypsin without EDTA, washed twice with assay buffer (Cayman Chemical), and pelleted. Cell pellets were fixed and stained using a Cell Cycle Phase Determination kit (Cayman Chemical) per manufacturer’s instructions. Cells were analyzed with flow cytometry (Sony SA3800 Spectral Cell Analyzer, Sony Biotechnology, USA).^37^

#### Western Blotting Assay

After the treatment of indicated concentrations of compounds, SAHA or tranylcypromine for 48 h, cells were washed with chilled PBS. After treatment, cells (5 × 10^6^) were harvested by scraping with RIPA buffer containing protease inhibitors and phosphatase inhibitor cocktail 3. Cell lysates were centrifuged at 14,000*g* for 30 min. For Western blot analysis, the amount of protein (40 μg) was separated by electrophoresis in a 12% polyacrylamide gel and transferred to a polyvinylidene difluoride (PVDF) membrane. For primary antibodies, antiacetyl-α-tubulin (Lys40; 1:1000 dilution), antiacetyl-Histone H3 (Lys9; 1:1000 dilution), anti-Histone H3K4me2 (Dimethyl Lys4; 1:1000 dilution), anti-PARP (1:500 dilution), anti-LSD1 (1:1000 dilution), anti-Caspase-3 (1:1000 dilution), anti-Caspase-9 (1:1000 dilution), anti-GAPDH (1:5000 dilution), and antibeta actin (1:1000 dilution) were used.^37^ For secondary antibodies, HRP-conjugated goat antimouse (1:10,000 dilution) and HRP-conjugated goat antirabbit (1:10,000 dilution) IgG antibodies were used. The detection of signal was done with an enhanced chemiluminescence detection kit and photographed by iBright FL1500 imaging systems (Invitrogen, Carlsbad, California, USA).

#### HDAC Enzymes and LSD1 Inhibition Assays

Enzyme inhibition assays were performed by the Reaction Biology Corporation, Malvern, Pennsylvania. (http://www.reactionbiology.com).

LSD1 inhibitory assay was also performed using the KDM1/LSD1 Activity Quantification/Colorimetric Assay Kit, as described earlier.^38^

#### Xenograft Model

To assess the antitumor effects of compound **2**, 4-week-old male Balb/c nude mice were subcutaneously injected with 1 × 10^7^ CRC cells (HCT-116). Once the tumor sizes reached 150 mm^3^, the mice were divided into four treatment groups, each comprising six mice. Compound **2**, formulated at specified dosages (50 or 100 mg/kg) in a vehicle consisting of 5% DMSO and 5% Cremophor EL in D5W (5% glucose), was administered through intraperitoneal injection (i.p.) once daily (q.d.). Irinotecan was employed as the reference drug, administered intraperitoneally weekly (qwk) for this experiment. Throughout the experiment, tumor size and body weight were measured twice a week, with tumor volume (mm^3^) calculated using the formula *LW*^2^/2 (*L* is the tumor length, and *W* is the tumor width). Tumor growth inhibition (TGI) was calculated by dividing the tumor volumes from treatment groups by those of the control groups, expressed as a percentage. Animal experiments adhered to relevant guidelines, ethical standards, and approved protocols by the Animal Use and Management Committee of Taipei Medical University (IACUC number: LAC-2017-0167).

#### Organoid Culture and Cell Proliferation Assay

CRC PDOs purchased from American Type Culture Collection (ATCC: HCM-CSHL-0257-C18) and cultured in Organoid Growth Kit 1A (ATCC: ACS-7100) supplemented with penicillin/streptomycin. PDOs are cultured by using its recommended protocol. Briefly, PDOs were processed and cultivated in rBM (growth factor reduced Matrigel) and were subcultured in rBM every 7 days.

PDOs were plated in 24-well plates and cultured for 3 days. After that, PDOs were treated with compound and cell viability assay was performed. Cell viability was measured by using Cell Counting Kit-8 (CCK-8, Dojindo) according to the manufacturers’ recommendations. Briefly, 5 μL of CCK-8 reagent was added in 100 μL medium and incubated for 2 h, 37 °C, 5% CO_2_, and subsequently, absorbance at 450 nm was measured by using SpectraMax iD5.

#### Immunoblot Analysis of Drug-Treated CRC PDOs

CRC PDOs were resuspended in Dispase-II by pipetting and spun down at 400*g* and 4 °C for 4 min, and then repeated twice for a total of three dissociation steps to remove the residual Matrigel. The harvested PDOs were lysed for 10 min at 4 °C in buffer A (10 mM HEPES, 1.5 mM MgCl_2_, 10 mM KCl, and 0.05% NP-40, pH 7.9) in the presence of protease and phosphatase inhibitors.^39,40^ The lysates were centrifuged again for 10 min at 4 °C at 14,000*g*. Subsequently, 40 μg of protein from the supernatant was resolved by SDS-PAGE and transferred onto PVDF membranes. The immunoblot analysis was then carried out as described in the earlier section on Western blotting assays.

#### Histological Analysis and Immunohistochemistry of Drug-Treated CRC PDOs

CRC PDOs were covered with 4% solution of formaldehyde for 10 min at room temperature. Next, CRC PDOs were scraped off the plates and gently plated in hot Histogel. Histogel was solidified at room temperature, embedded in paraffin, and sectioned. CRC PDOs were cut at a thickness of 3.5 μm for staining. CRC PDOs were deparaffinized in clear tissue and hydrated in ethanol solutions at decreasing concentrations before further treatment. For immunohistochemical stainings, heat-induced antigen retrieval was performed at 95 °C for 15 min in 10 mM sodium citrate, pH 6.0 (for Caspase-3, Caspase-7, Histone H3K4me2, and LSD1), or at 95 °C for 20 min in Tris–EDTA buffer (10 mM Tris base, 1 mM EDTA, 0.05% Tween 20, pH 9.0) (for Caspase-9). Endogenous peroxidase activity was blocked by incubation in 1% (v/v) hydrogen peroxide for 15 min. The sections were then incubated overnight in Shandon racks (Thermo Shandon) at 4 °C with the primary antibodies (100 μL/section) diluted in Antibody Diluent to the following concentrations: anti-Caspase-3 (1:1000 dilution), anti-Caspase-7 (1:200 dilution), anti-Caspase-9 (1:300 dilution), anti-Histone H3K4me2 (Dimethyl Lys4) (1:100 dilution), and anti-LSD1 (1:200 dilution). Envision horseradish peroxidase-labeled antirabbit IgG (K4003, Dako) was used for detection (100 μL/section) by incubating 45 min at room temperature, followed by development with the Vector NovaRED substrate kit, peroxidase (HRP) for 9 min as specified by the manufacturer. The sections were then counterstained with Mayer’s hematoxylin for 30 s. The immunoperoxidase stained sections were finally dehydrated in ethanol solutions before mounting in Glas Tissue-Mount. Images were acquired using the TissueGnostics Axio Observer Z1 microscope (TissueGnostics GmbH, Vienna, Austria) at 20× with high-precision autofocus. Captured images were viewed with HistoQuest (TissueGnostics, Vienna, Austria).

## Supporting Information Available

^1^H NMR spectra for compounds **1**–**10**; ^13^C NMR spectra for compounds **1**–**10**; HPLC purity data for compounds **1**–**10**; and results of in vivo pharmacokinetic study of compound **2** (PDF)
Characterization data of compounds (CSV)

## Abbreviations

AKT: AKT serine/threonine kinase
AML: acute myeloid leukemia
BRAF: serine/threonine-protein kinase B-raf
CDH-1: cadherin 1
CRC: colorectal cancer
G0/G1 phase: gap 0 and gap 1 phase
G2/M phase: gap 2 and mitosis phase
GI_50_ values: growth inhibition 50%
HDACs: histone deacetylases
H3K4me1/2: mono- and dimethyl lysine 4 of histone 3
H3K9me1/2: mono- and dimethyl lysine 9 of histone 3
KDM1A: lysine-specific demethylase 1A
IC_50_: half maximal inhibitory concentration
LSD1: lysine-specific demethylase 1
PDOs: patient-derived organoids
PIK3CA: phosphatidylinositol-4,5-bisphosphate 3-kinase catalytic subunit alpha
rBM: growth factor reduced Matrigel
SAHA: suberoylanilide hydroxamic acid (vorinostat)
S phase: synthesis phase
TGI: tumor growth inhibition rate
Tra: tranylcypromine
