# Supplementary material for: Syntheses of LSD1/HDAC Inhibitors with Demonstrated Efficacy against Colorectal Cancer: In Vitro and In Vivo Studies Including Patient-Derived Organoids
Source: J Med Chem. 2024 Sep 25;67(19):17207–25. doi: 10.1021/acs.jmedchem.4c01098 (PMC11472331; doi:10.1021/acs.jmedchem.4c01098)
Supplement: Supplementary file 1 — jm4c01098_si_001.pdf [file jm4c01098_si_001.pdf]

Supporting information

Syntheses of LSD1/HDAC Inhibitors with Demonstrated Efficacy Against Colorectal Cancer: *In Vitro* and *In Vivo* Studies Including Patient-Derived Organoids

Po-Yu Chou[a#], Mei-Jung Lai[b#], Kelvin K. Tsai[c,d], Li-Hsin Cheng[d], Yi-Wen Wu[e], Mei-Chuan Chen[a, b, g, h, i], Shiow-Lin Pan[b, e, f, g, j], Hsiu-O Ho[a], Kunal Nepali[a, b, g]*, Jing-Ping Liou [a, b, f, g]*

[#]Contributed equally to this work.

[a] School of Pharmacy, College of Pharmacy, Taipei Medical University, Taipei 110, Taiwan.

[b] TMU Research Center for Drug Discovery, Taipei Medical University, Taipei 110, Taiwan.

[c] Laboratory of Advanced Molecular Therapeutics, Graduate Institute of Clinical Medicine, College of Medicine, Taipei Medical University, Taipei 110, Taiwan.

[d] Organoids Technology Core, Taipei Medical University, Taipei 110, Taiwan

[e] Graduate Institute of Cancer Biology and Drug Discovery, College of Medical Science and Technology, Taipei Medical University, Taipei 110, Taiwan

[f] TMU Research Center of Cancer Translational Medicine, Taipei 110, Taiwan

[g] Ph.D. Program in Drug Discovery and Development Industry, College of Pharmacy, Taipei Medical University, Taipei 110, Taiwan.

[h] Clinical Drug Development of Herbal Medicine, College of Pharmacy, Taipei Medical University, Taipei 110, Taiwan.

[i] Traditional Herbal Medicine Research Center of Taipei Medical University Hospital, Taipei 110, Taiwan

[j] Ph.D. Program for Cancer Molecular Biology and Drug Discovery, College of Medical Science and Technology, Taipei Medical University, Taipei 110, Taiwan

* Corresponding author. Nepali K.: Phone: 886-2-2736-1661 ext. 6134; E-mail: nepali@tmu.edu.tw Liou J. P.: Phone: 886-2-2736-1661 ext 6130. E-mail: jpl@tmu.edu.tw

Contents:

1. ^{1}H NMR Spectra for compounds **1-10**……………………………………………………..S2-6
2. ^{13}C NMR Spectra for compounds **1-10**…………………………………………………...S7-11
3. HPLC purity data for compounds **1-10**...S12-16
4. Results of *in-vivo* pharmacokinetic study of compound 2……………………………S17

^{1}H spectrum of compound 1

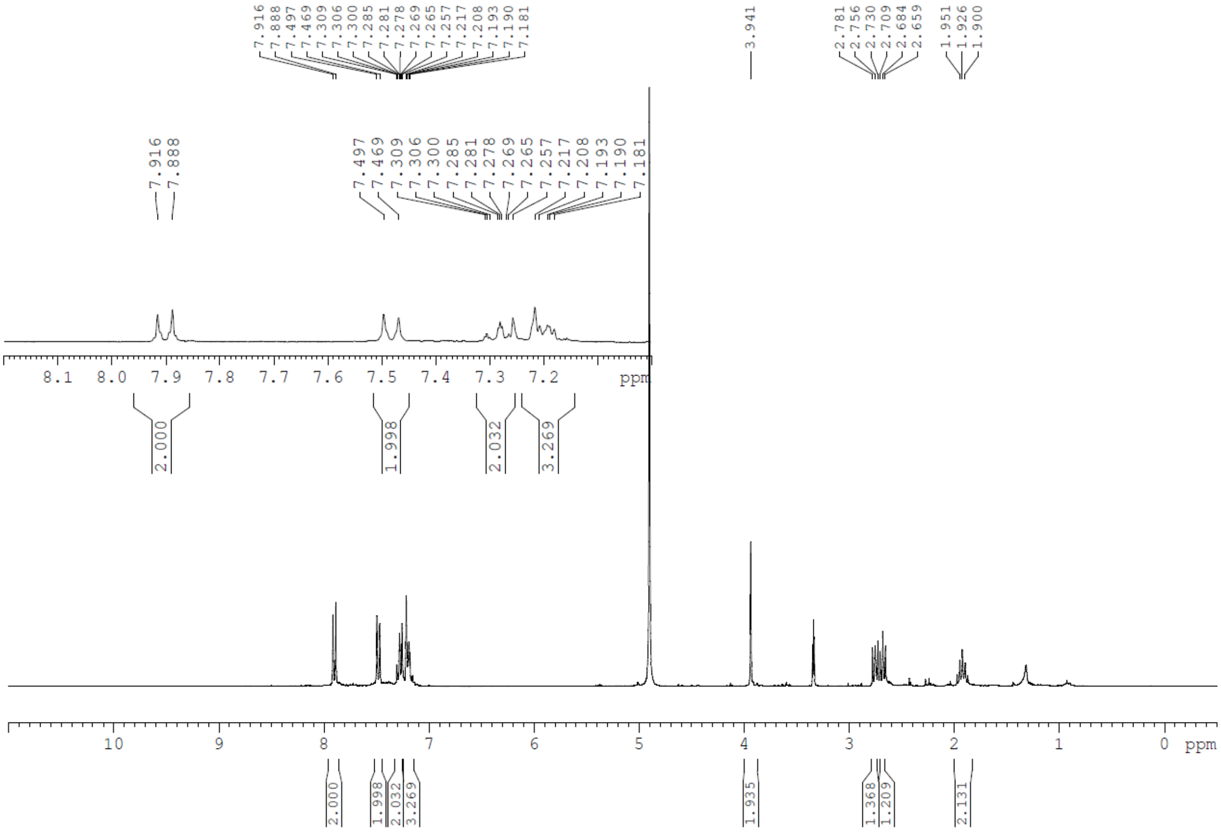

^{1}H spectrum of compound 2

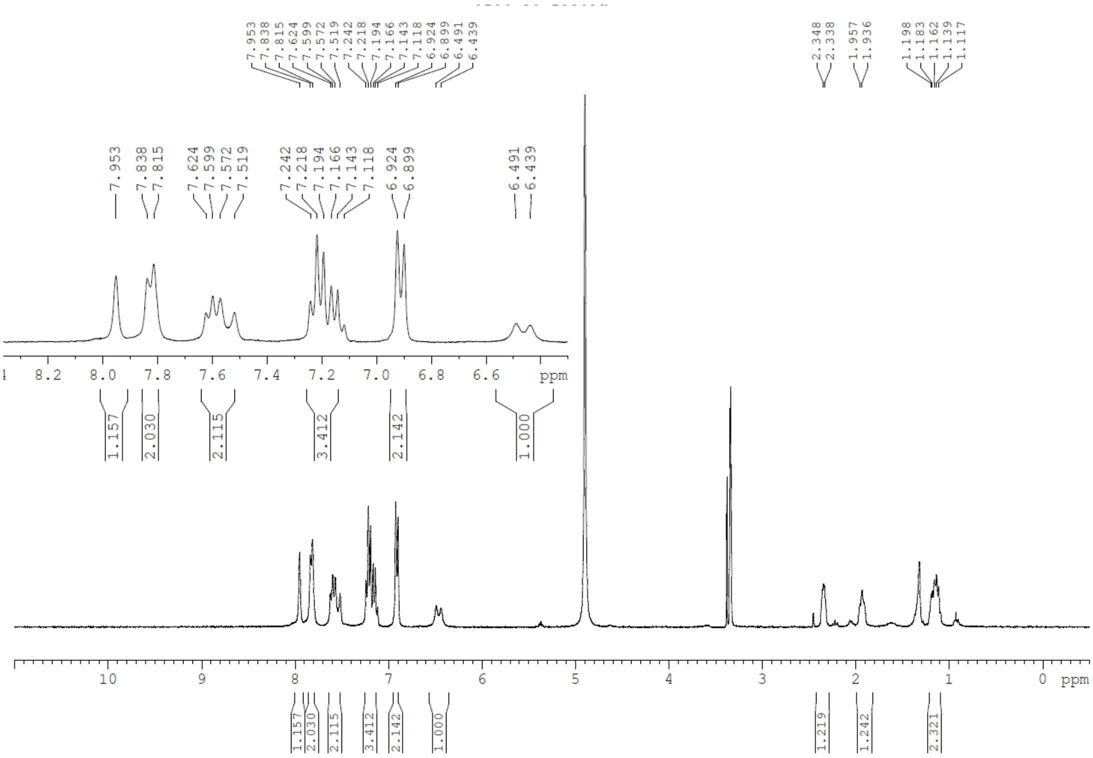

^{1}H spectrum of compound 3

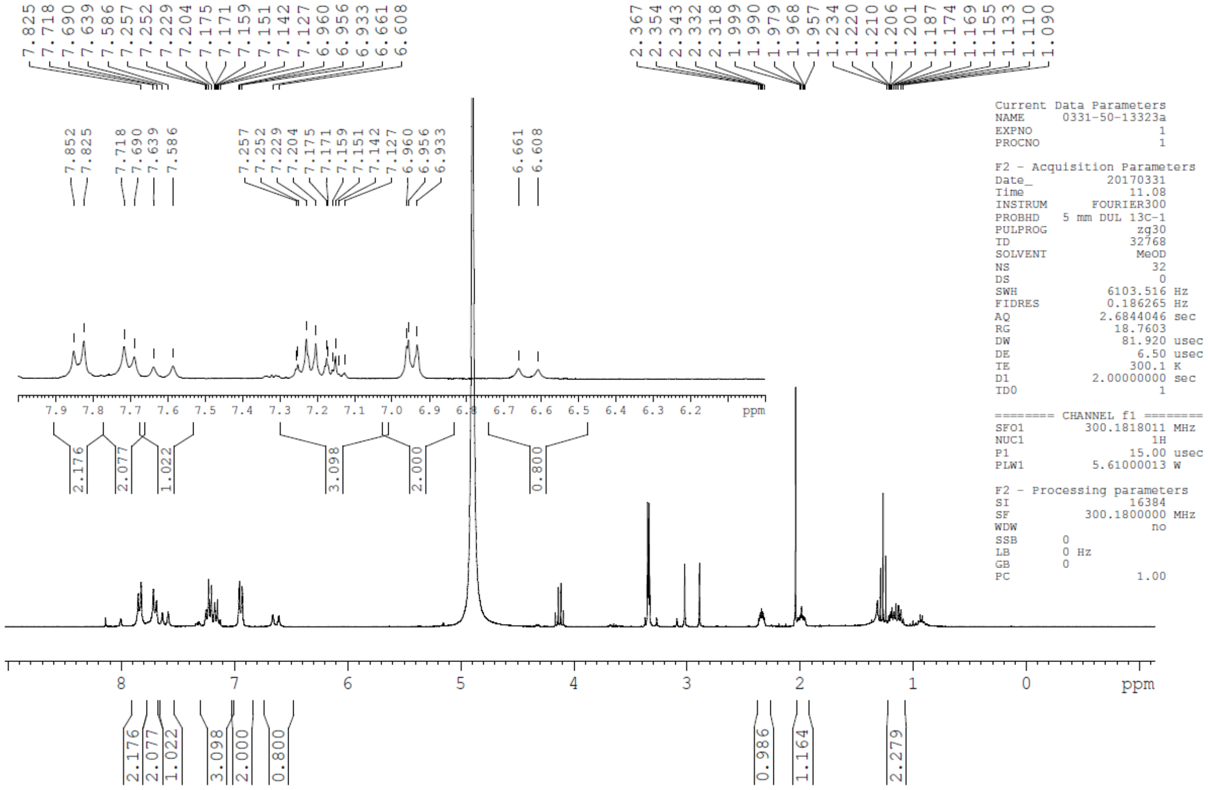

^{1}H spectrum of compound 4

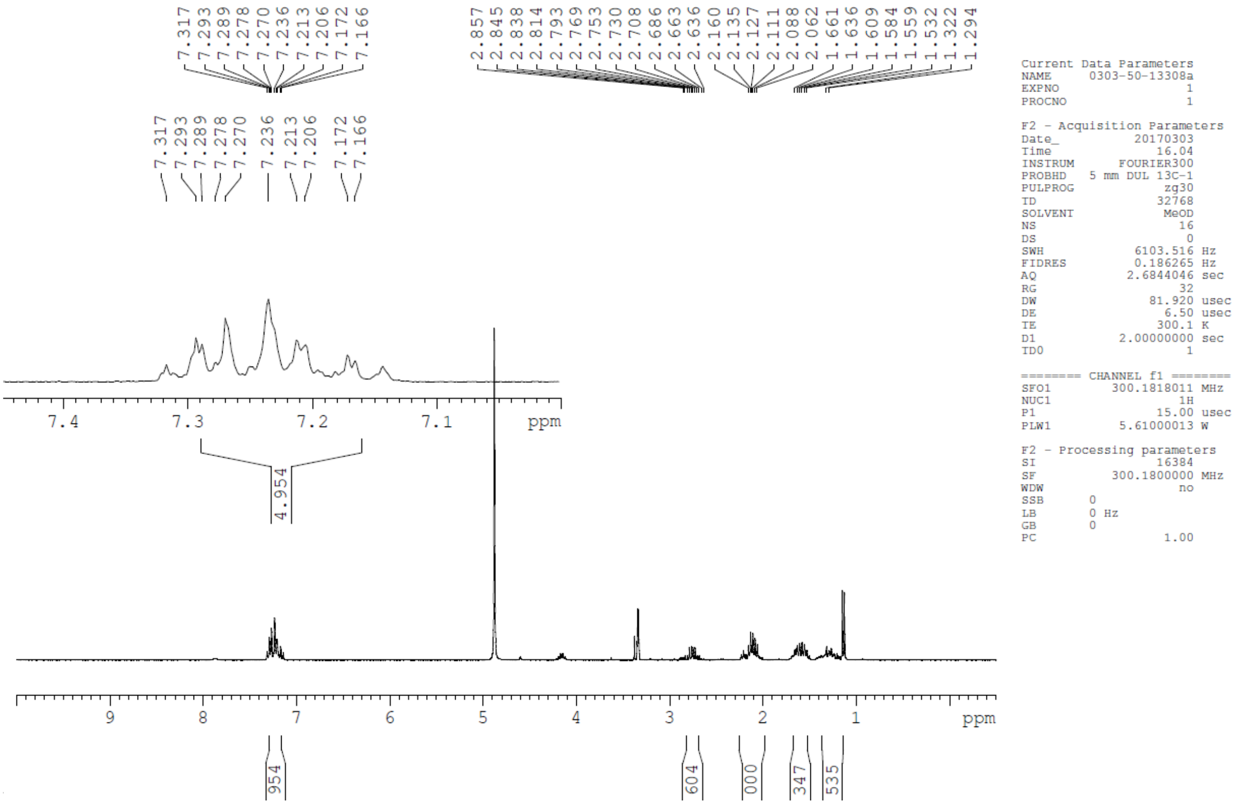

^{1}H spectrum of compound 5

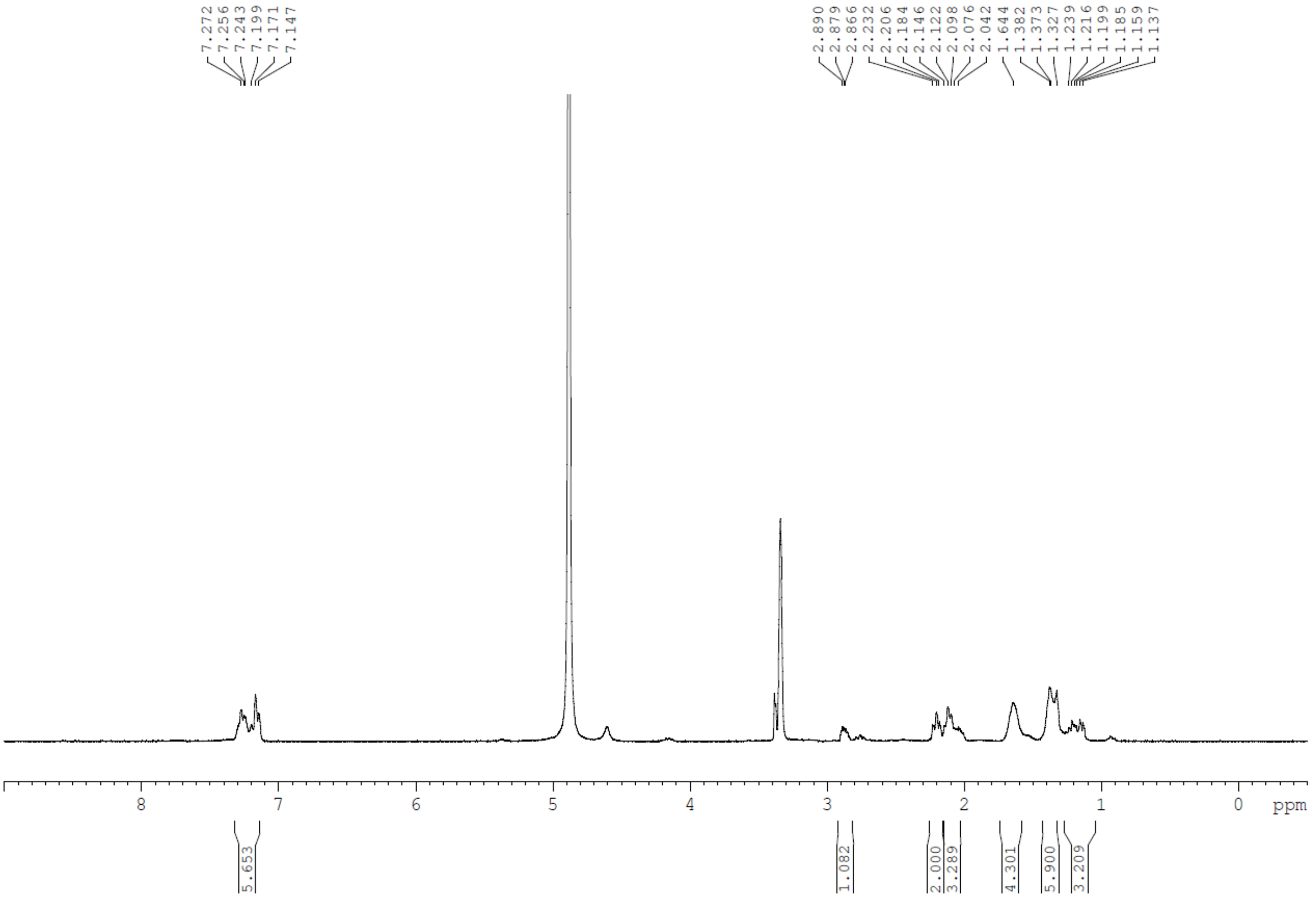

^{1}H spectrum of compound 6

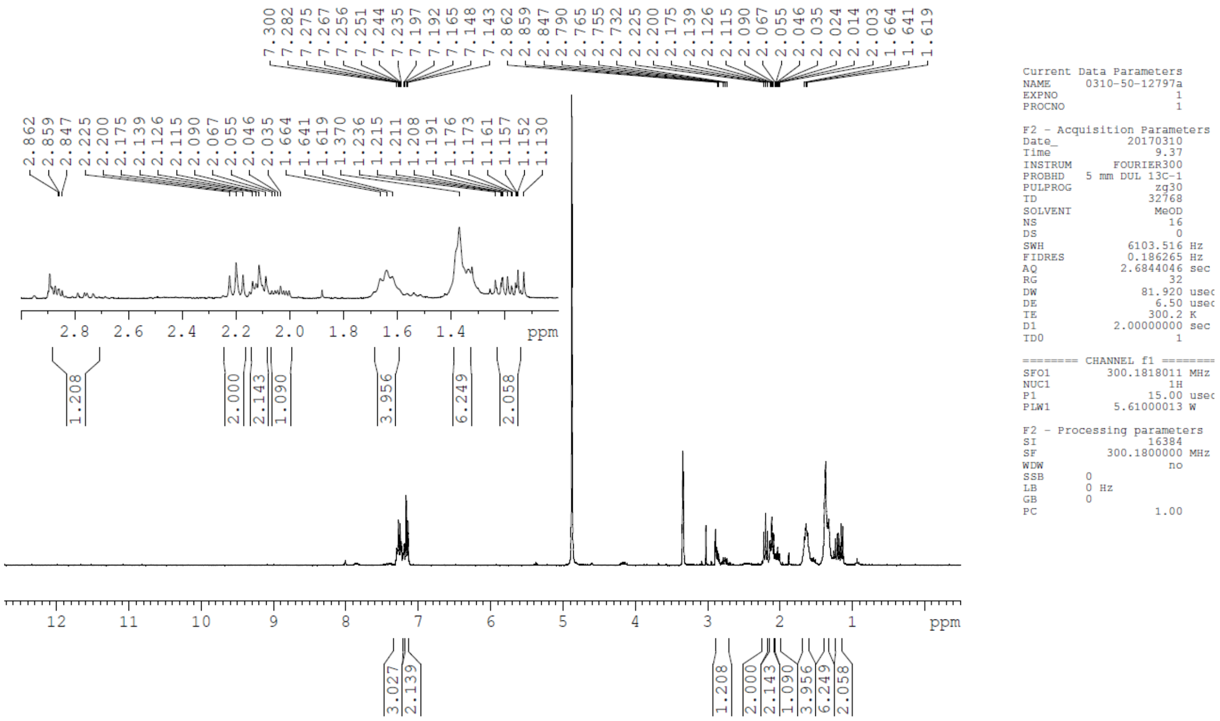

^{1}H spectrum of compound 7

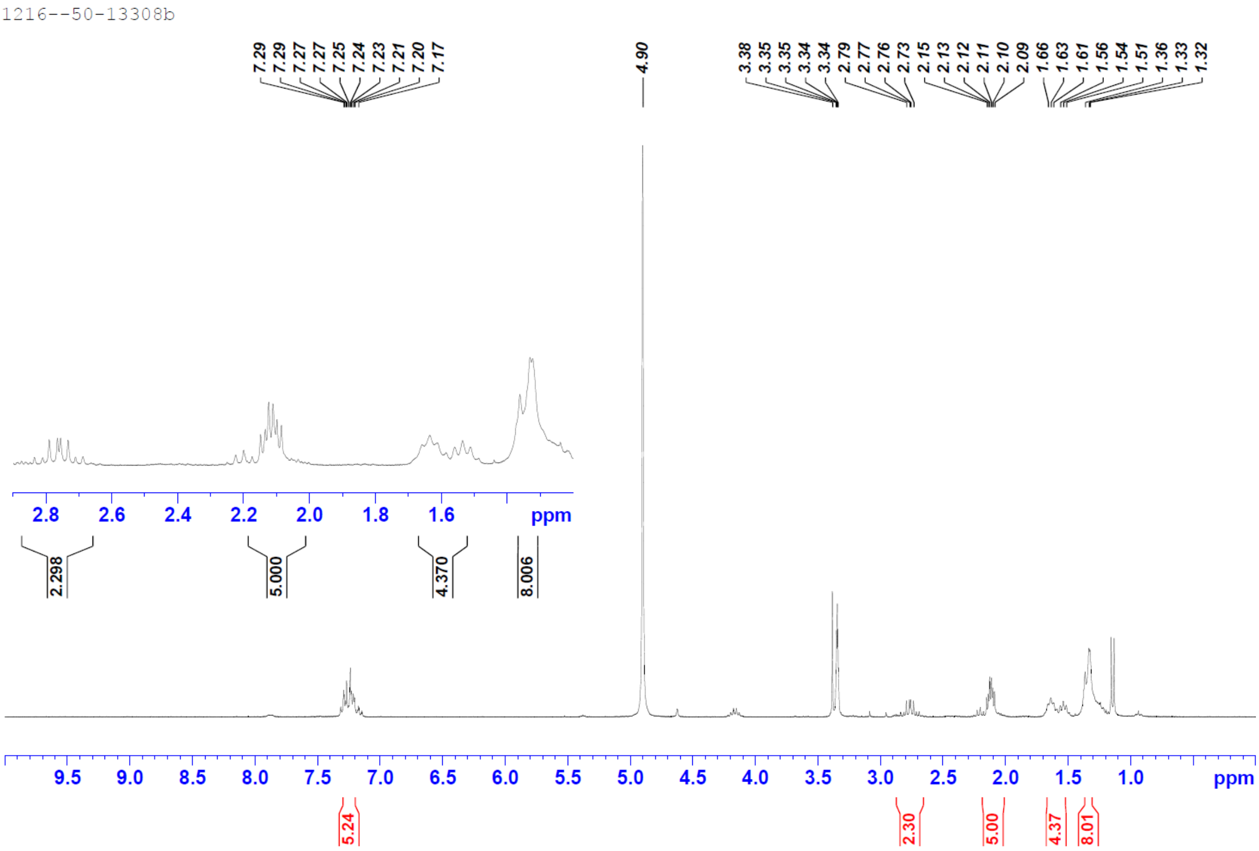

^{1}H spectrum of compound 8

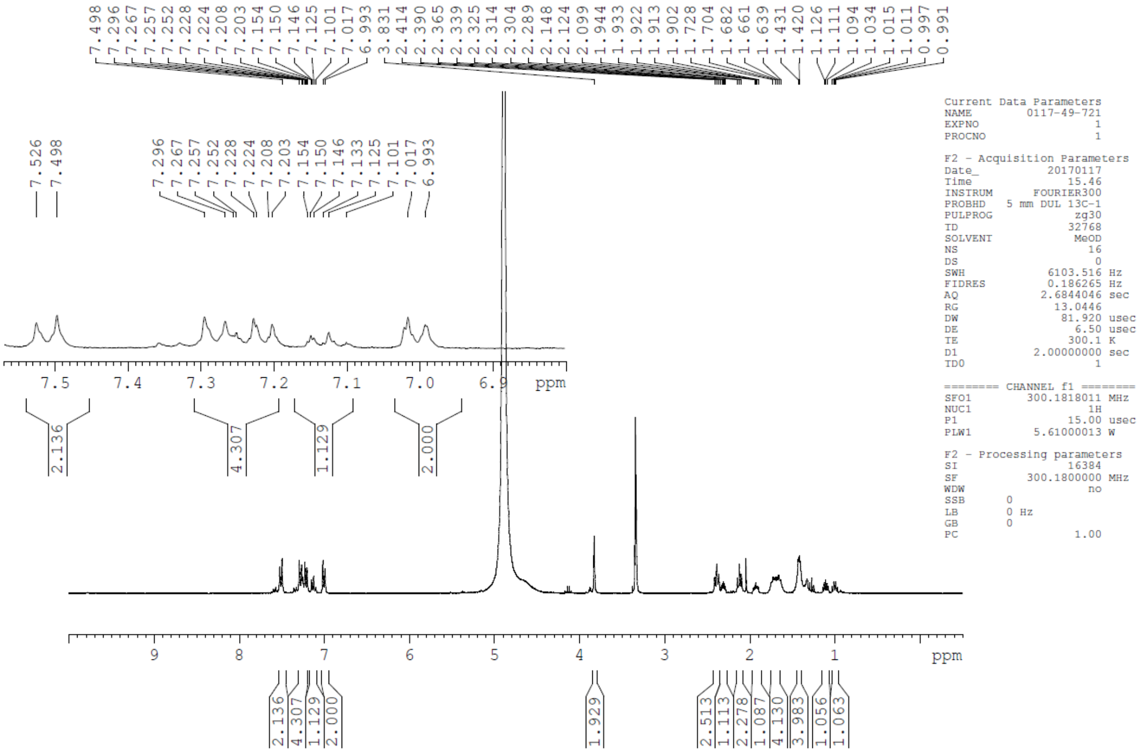

^{1}H spectrum of compound 9

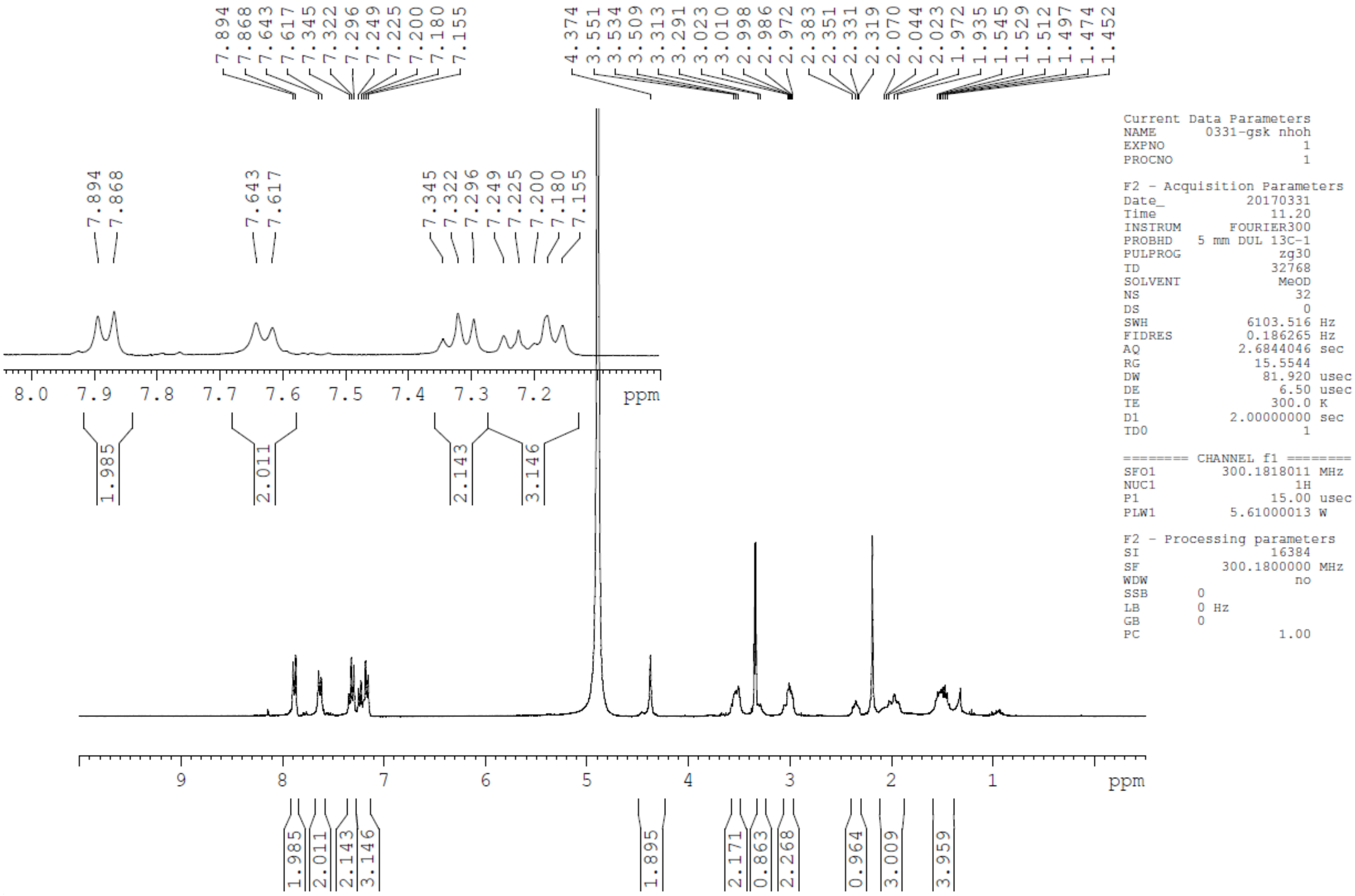

^{1}H spectrum of compound 10

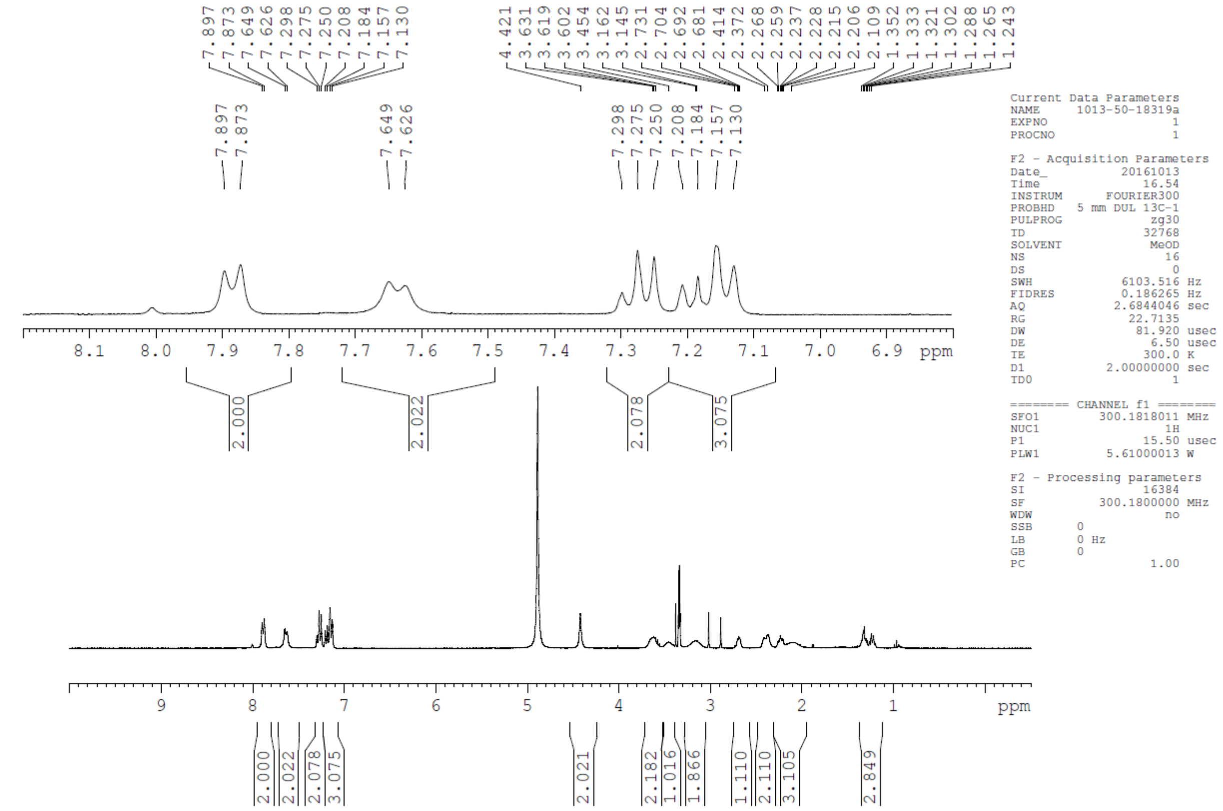

^{13}C spectrum of compound 1

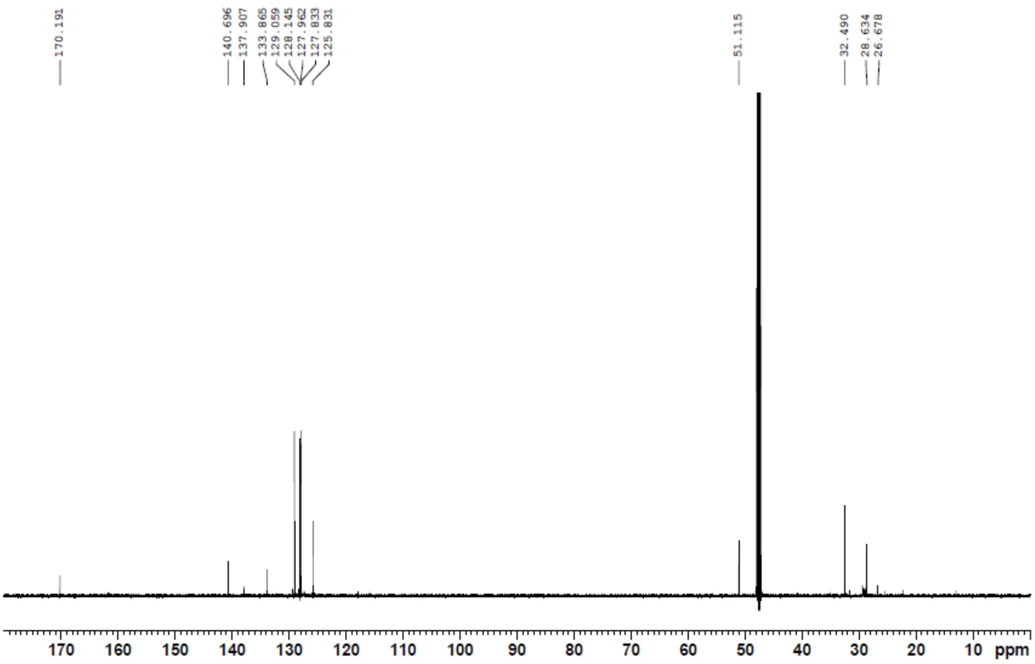

^{13}C spectrum of compound 2

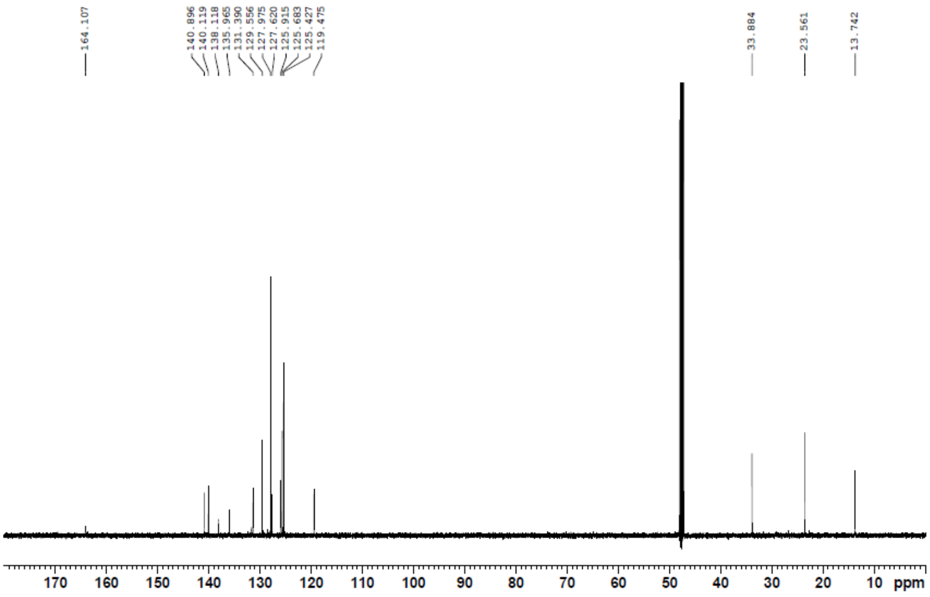

^{13}C spectrum of compound 3

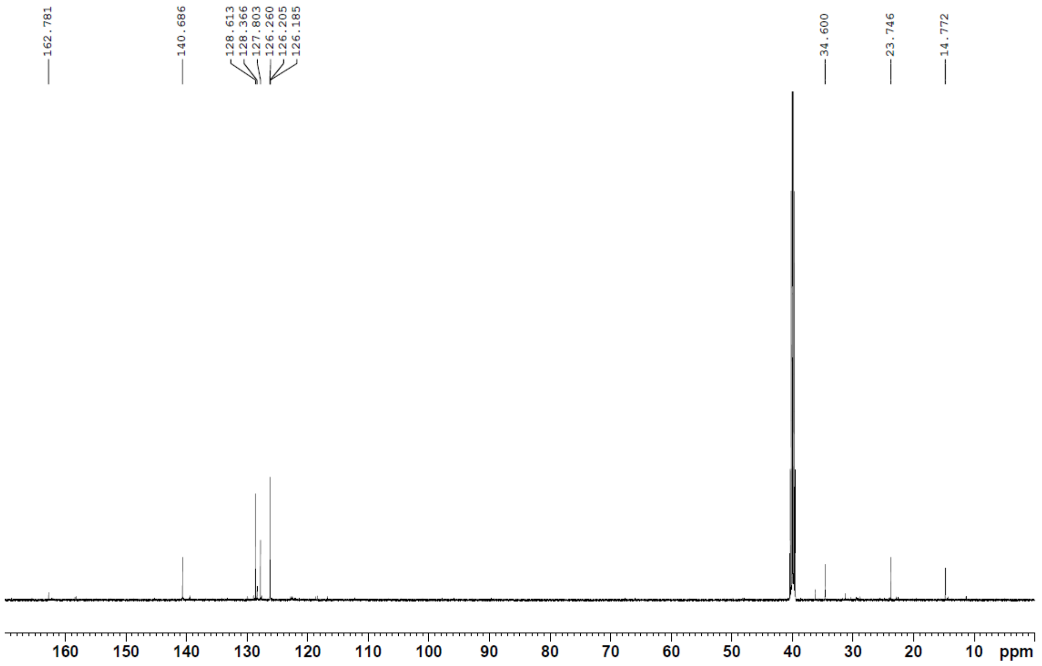

^{13}C spectrum of compound 4

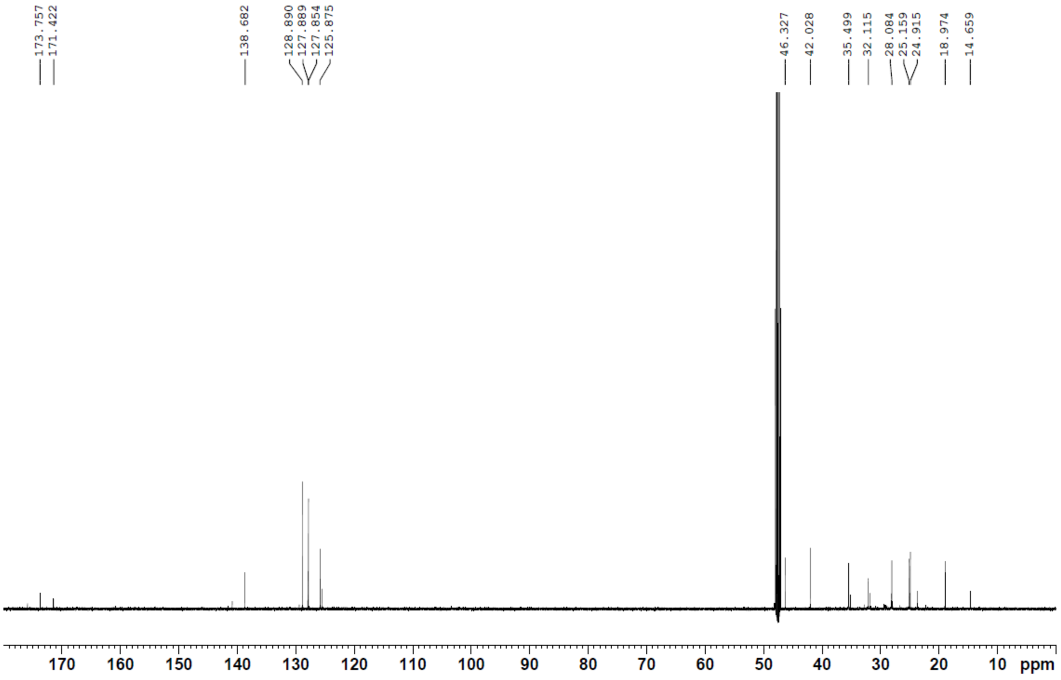

^{13}C spectrum of compound 5

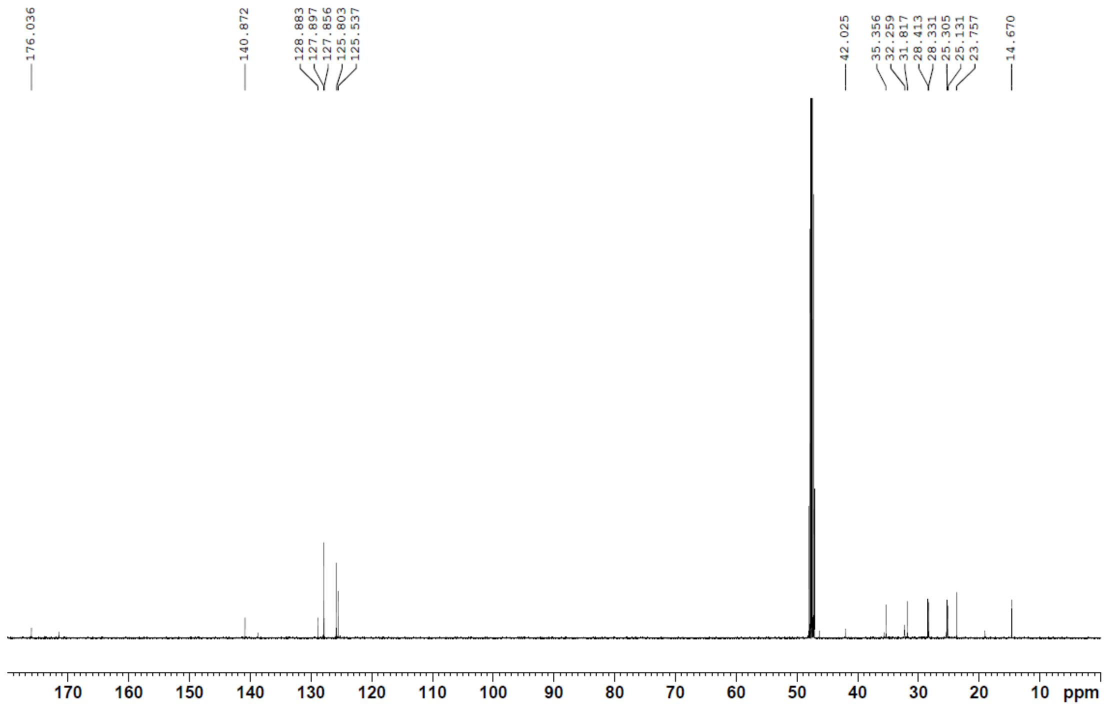

^{13}C spectrum of compound 6

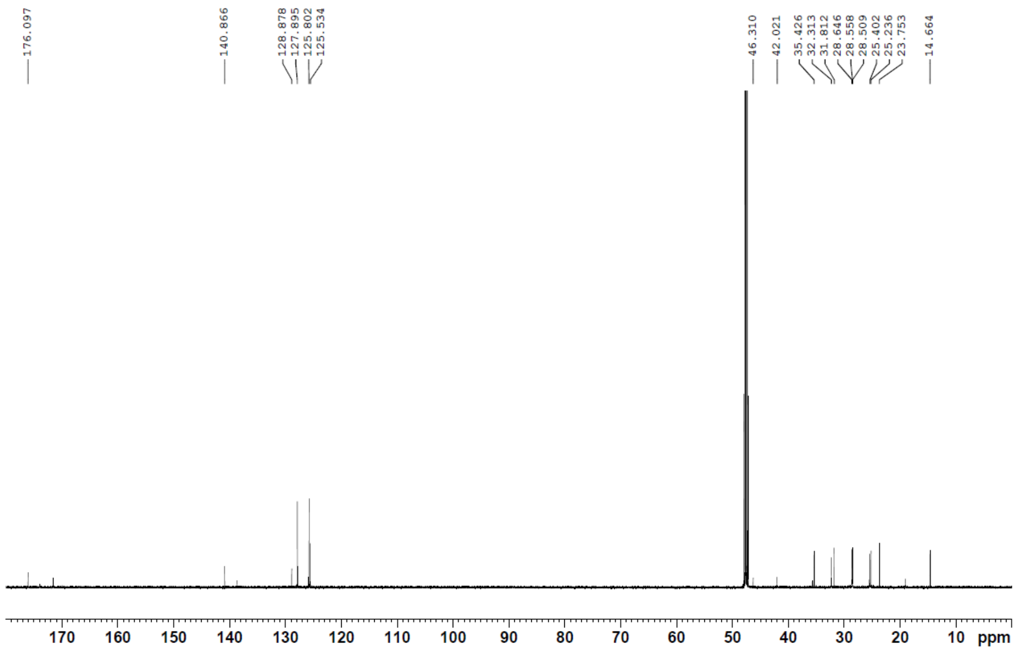

^{13}C spectrum of compound 7

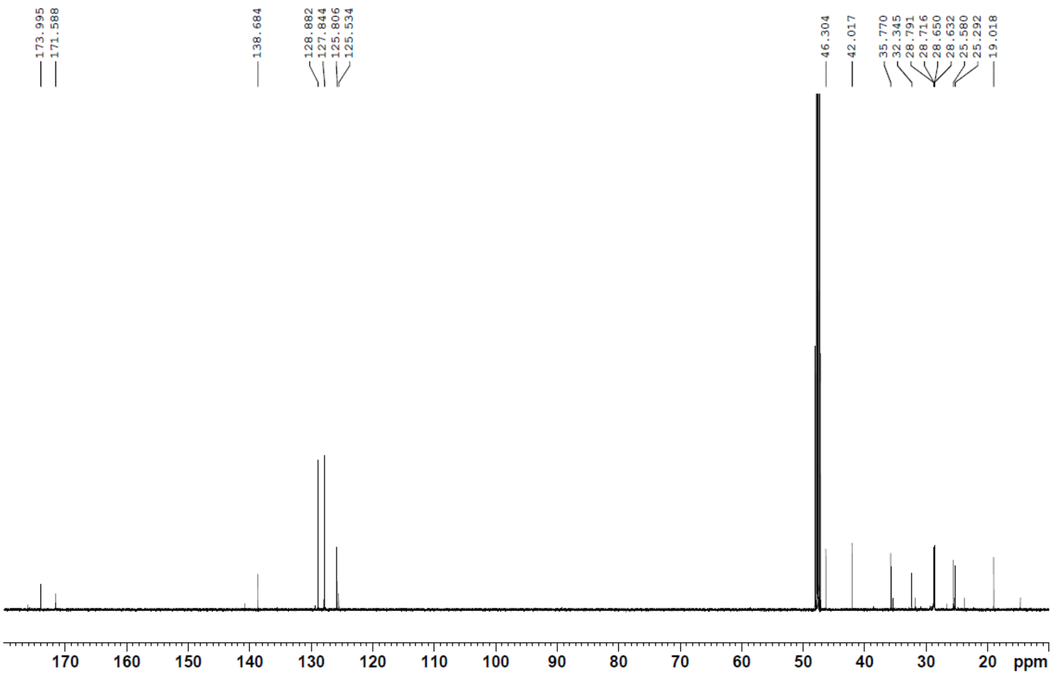

^{13}C spectrum of compound 8

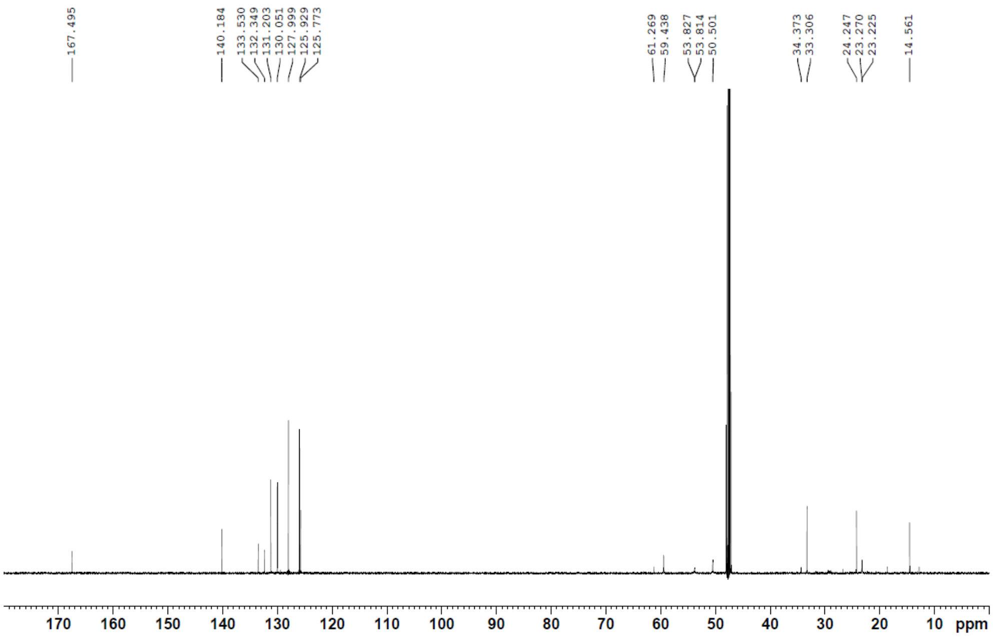

^{13}C spectrum of compound 9

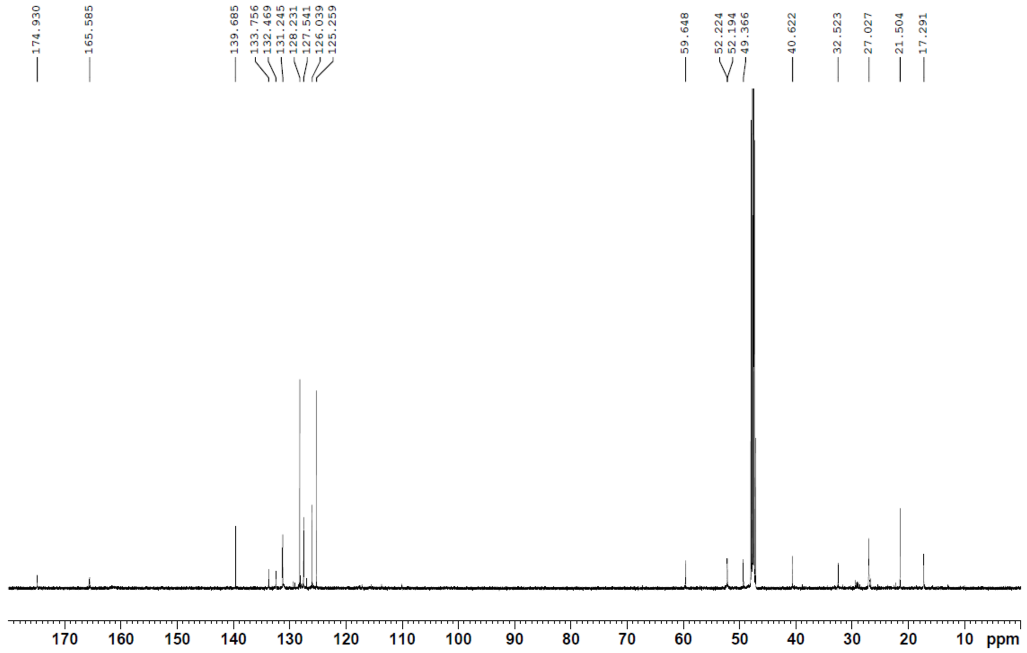

^{13}C spectrum of compound 10

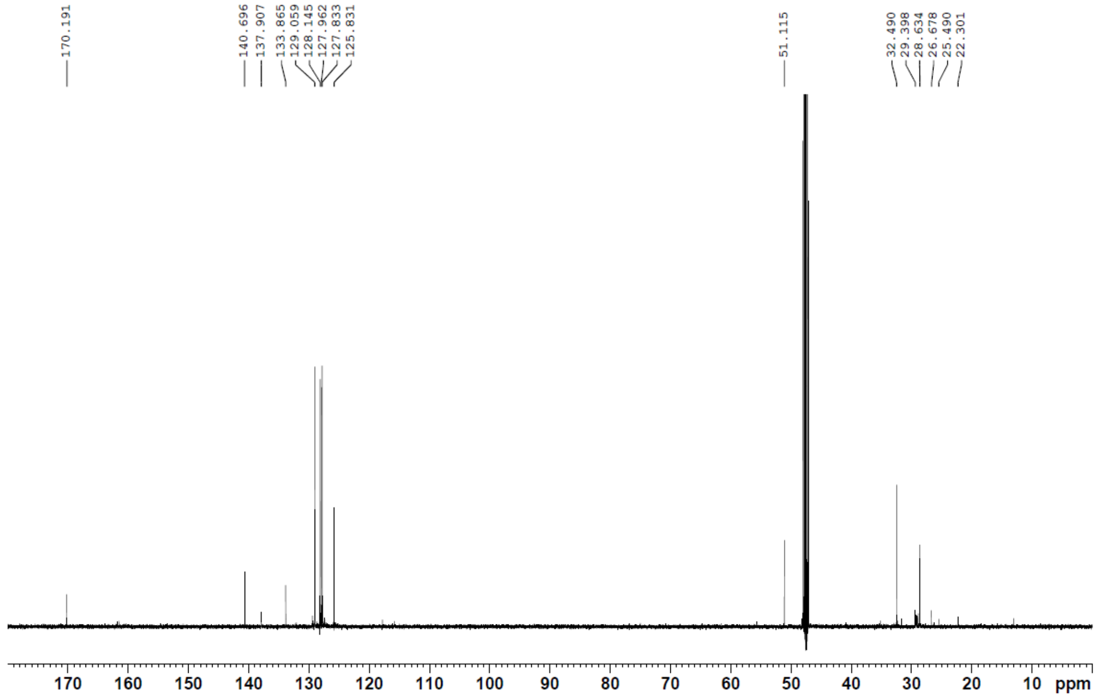

HPLC Chromatogram of compound 1

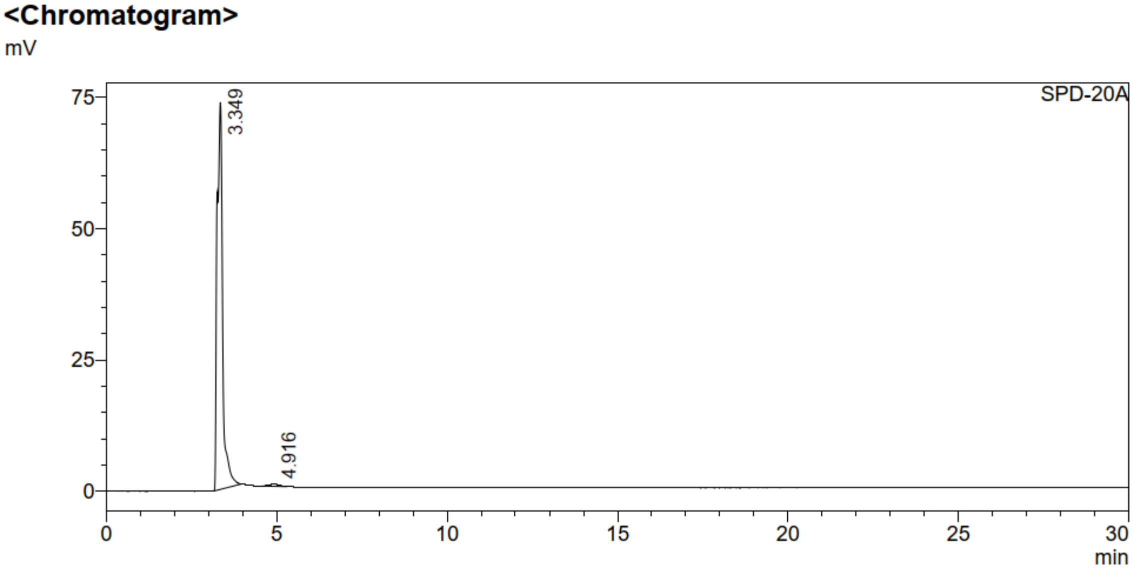

Peak Table

SPD-20A

Peak#	Ret. Time	Area	Height	Area%
1	3.349	832723	73679	98.975
2	4.916	8622	374	1.025
Total		841345	74052	100.000

HPLC Chromatogram of compound 2

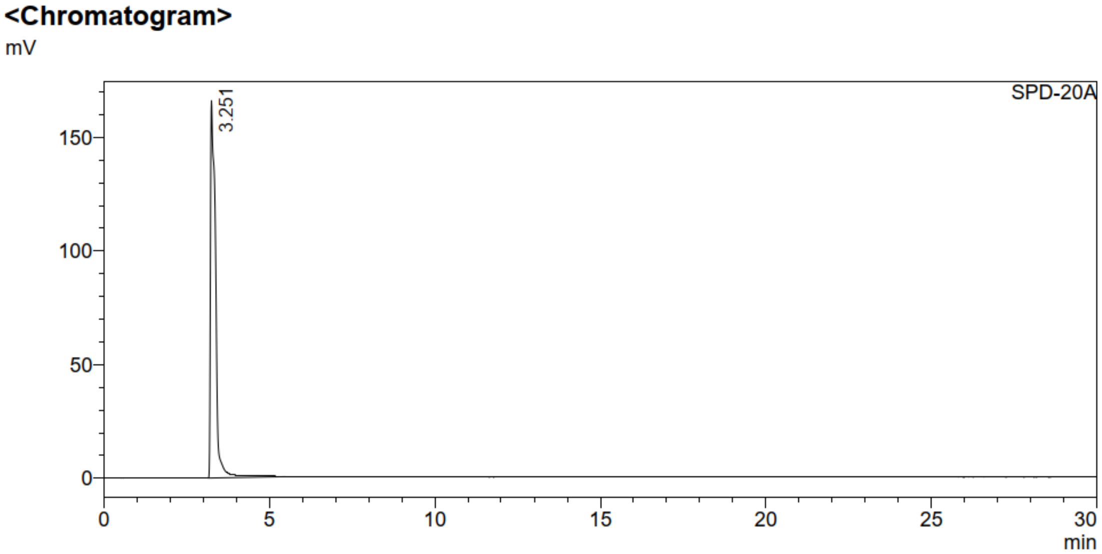

Peak Table

SPD-20A

Peak#	Ret. Time	Area	Height	Area%
1	3.251	1785637	166160	100.000
Total		1785637	166160	100.000

HPLC Chromatogram of compound 3

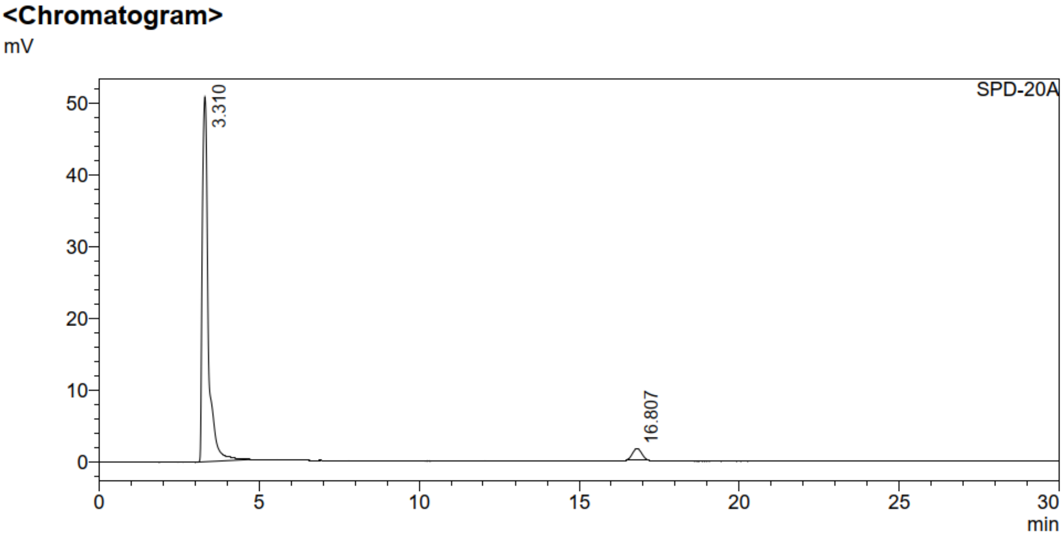

Peak Table

SPD-20A

Peak#	Ret. Time	Area	Height	Area%
1	3.310	666846	50763	95.465
2	16.807	31675	1595	4.535
Total		698521	52358	100.000

HPLC Chromatogram of compound 4

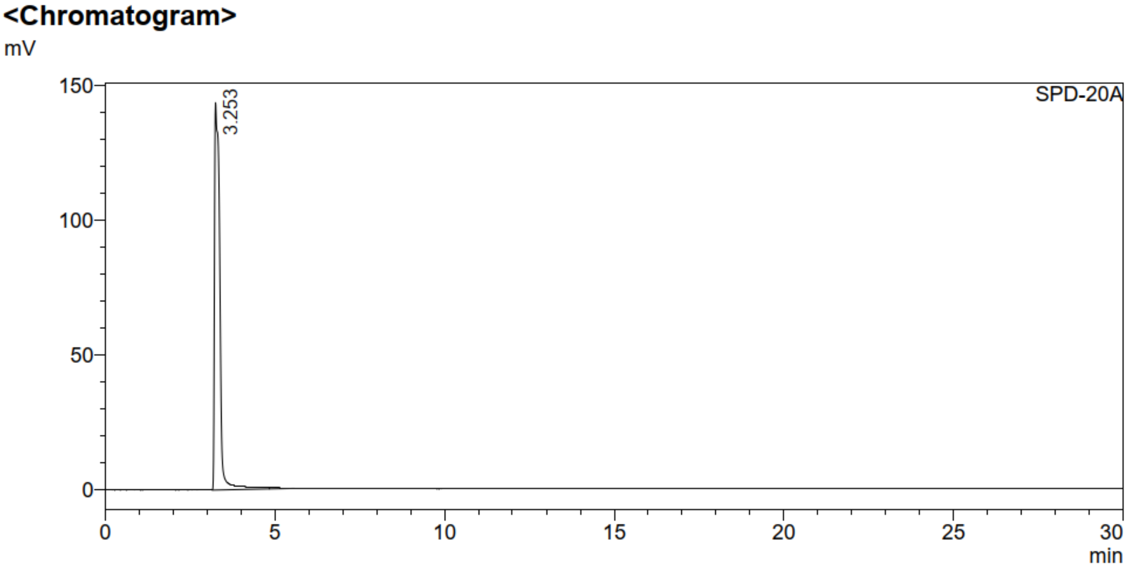

Peak Table

SPD-20A

Peak#	Ret. Time	Area	Height	Area%
1	3.253	1567054	143654	100.000
Total		1567054	143654	100.000

HPLC Chromatogram of compound 5

<Chromatogram>

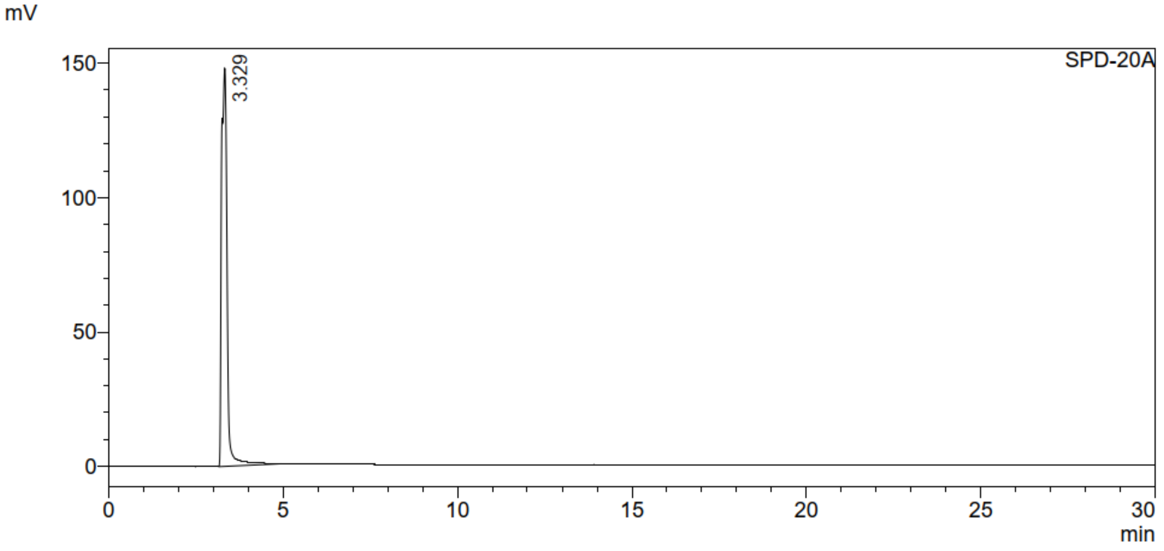

Peak Table

SPD-20A

Peak#	Ret. Time	Area	Height	Area%
1	3.329	1652937	147959	100.000
Total		1652937	147959	100.000

HPLC Chromatogram of compound 6

<Chromatogram>

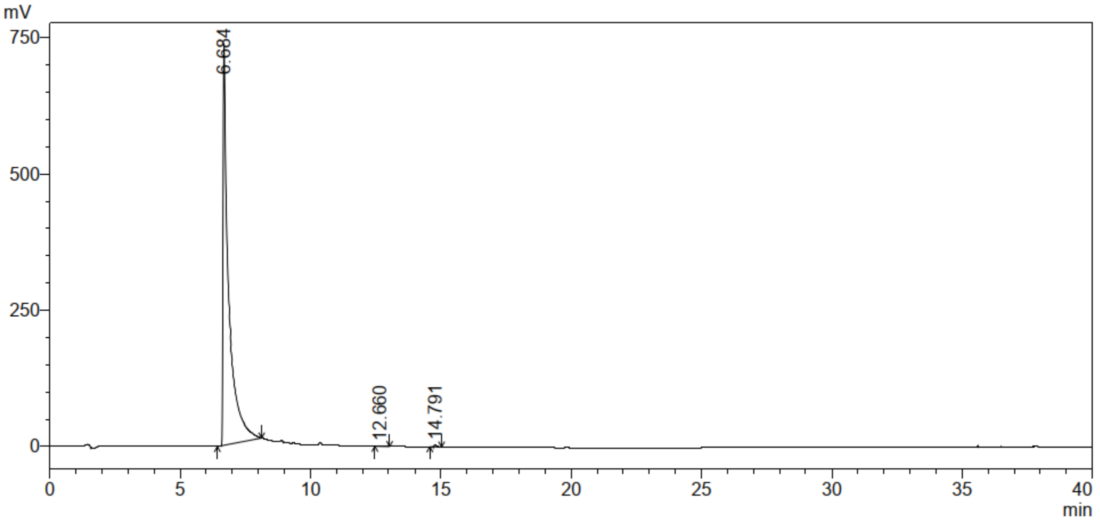

<Peak Table>

SPD-20A

Peak No.	Ret. Time	Area	Area%
1	6.684	10837843	99.697
2	12.660	7917	0.073
3	14.791	25053	0.230
Total		10870814	100.000

HPLC Chromatogram of compound 7

<Chromatogram>

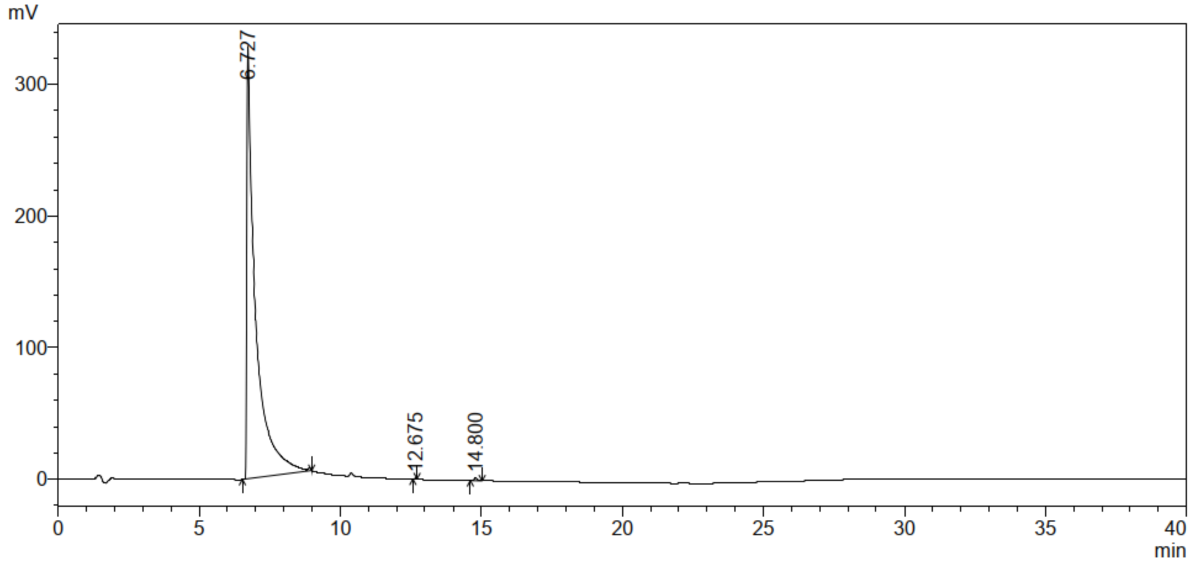

<Peak Table>

SPD-20A

Peak No.	Ret. Time	Area	Area%
1	6.727	7008320	99.746
2	12.675	2301	0.033
3	14.800	15523	0.221
Total		7026143	100.000

HPLC Chromatogram of compound 8

<Chromatogram>

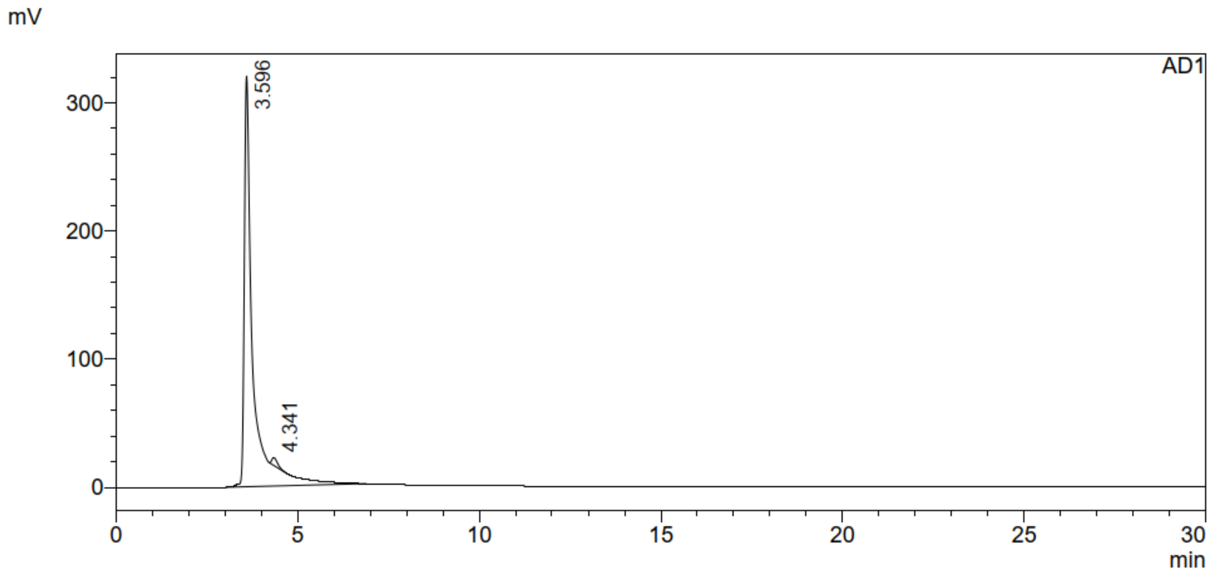

Peak Table

AD1

Peak#	Ret. Time	Area	Height	Area%
1	3.596	5279818	319872	98.760
2	4.341	66269	6116	1.240
Total		5346087	325988	100.000

HPLC Chromatogram of compound 9

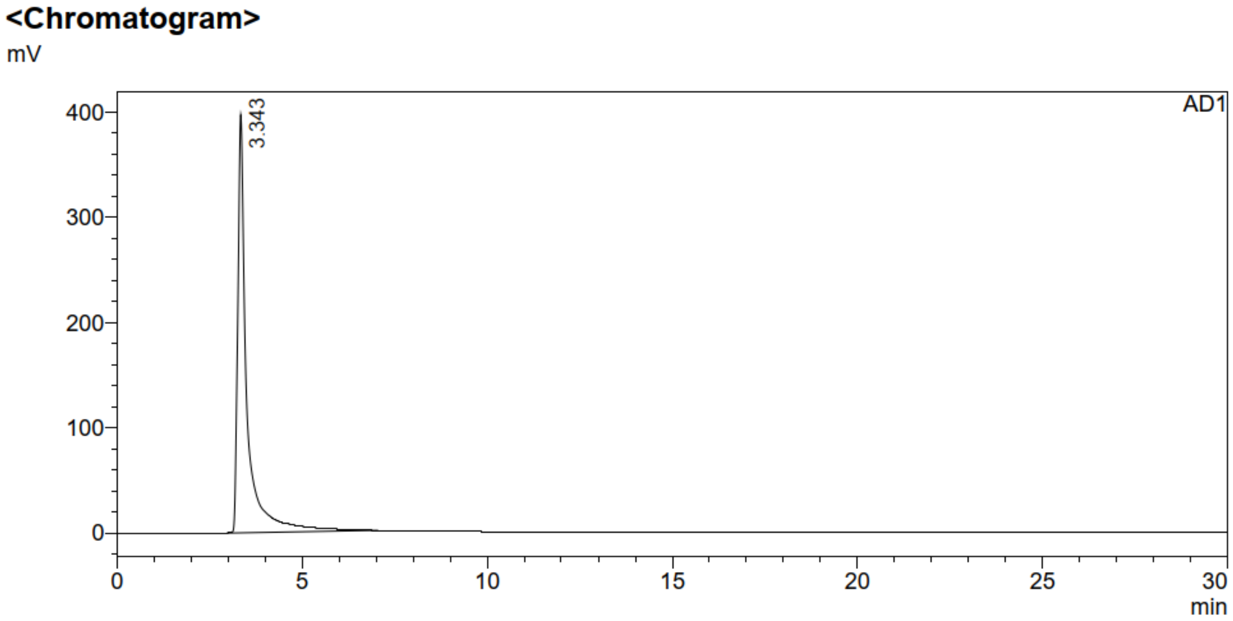

Peak Table

AD1

Peak#	Ret. Time	Area	Height	Area%
1	3.343	6826529	397177	100.000
Total		6826529	397177	100.000

HPLC Chromatogram of compound 10

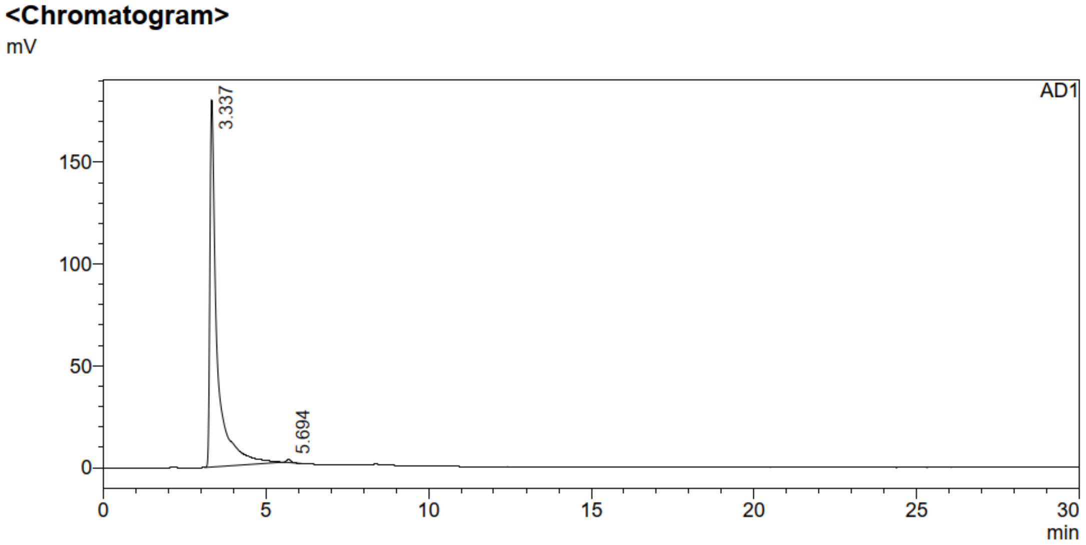

Peak Table

AD1

Peak#	Ret. Time	Area	Height	Area%
1	3.337	2792208	180144	99.440
2	5.694	15723	1599	0.560
Total		2807931	181743	100.000

***In-vivo* pharmacokinetic study of compound 2**

The *in-vivo* pharmacokinetic study of compound **2** was conducted in Male Sprague-Dawley rats. For this study, Male Sprague-Dawley rats, weighing 445-538 g each, were obtained week from BioLASCO, Taiwan Co., Ltd., Ilan, Taiwan. The rats were quarantined for one week prior to the experiment. Animals were surgically implanted with a jugular-vein cannula one day before dosing. The IV dose of 2 mg/kg and oral dose of 20 mg/kg (formulated in DMA/PEG, 20%/80%, v/v) were given to group of three rats each. The volume of dosing solution administered was adjusted according to the body weight recorded before dose administration. At 0 (immediately before dosing), 0.03, 0.08, 0.25, 0.5,1, 2, 4, 6, 8, and 24 hr after dosing, a blood sample (~150 µL) was withdrawn from each animal via the jugular-vein cannula and stored in ice (0-4 ^{o}C). Plasma was separated from the blood by centrifugation and stored in a freezer (-20 ^{o}C).

The results of the study are presented in **Table S1**.

Table S1. Results of *in-vivo* pharmacokinetic study of compound **2**[a]

PK profiles[b]	Rat	
	i.v. at 2 mg/kg	po at 20 mg/kg
$AUC_{0\text{-inf}}$ (ng/mL∗ hr)	1033 ± 568	82 ± 30
$T_{1/2}$ (hr)	11.4 ± 3.8	2.9 ± 1.5
C_{max} (ng/mL)	—	16.5 ± 1.1
T_{max} (hr)	—	1.1 ± 0.9
CL (mL/min/kg)	40 ± 30	—
V_{ss} (L/kg)	16 ± 5	—
F (%)	—	0.8

[a] Values are expressed as the mean of at least three independent experiments. [b]AUC, area under curve. *CL*, clearance. *F*, oral bioavailability. V_{ss}, volume at steady state. $T_{1/2}$, plasma half-life.
